# miR-3940-5p reduces amyloid β production via selectively targeting *PSEN1*

**DOI:** 10.3389/fnagi.2024.1346978

**Published:** 2024-03-04

**Authors:** Yanmei Qi, Xu Wang, Xihan Guo

**Affiliations:** ^1^School of Life Sciences, The Engineering Research Center of Sustainable Development and Utilization of Biomass Energy, Yunnan Normal University, Kunming, Yunnan, China; ^2^Yeda Institute of Gene and Cell Therapy, Taizhou, Zhejiang, China

**Keywords:** miRNAs, SH-SY5Y cells, Alzheimer’s disease, *PSEN1*, Aβ

## Abstract

Alzheimer’s disease (AD) is a progressive neurodegenerative disorder characterized by the accumulation of amyloid beta (Aβ) in brain. Mounting evidence has revealed critical roles of microRNAs (miRNAs) in AD pathogenesis; however, the miRNAs directly targeting *presenilin1* (*PSEN1*), which encodes the catalytic core subunit of γ-secretase that limits the production of Aβ from amyloid precursor protein (APP), are extremely understudied. The present study aimed to identify miRNAs targeting *PSEN1* and its effect on Aβ production. This study first predicted 5 candidate miRNAs that may target *PSEN1*,through websites such as TargetScan, miRDB, and miRwalk. Subsequently, the targeting specificity of the candidate miRNAs towards PS1 was validated using dual-luciferase reporter assays. To investigate the regulatory effect of miR-3940-5p on gene expression based on its targeting of PS1, miR-3940-5p mimics or inhibitors were transiently transfected into SH-SY5Y cells. Changes in *PSEN1* transcription and translation in the tested cells were detected using RT-qPCR and Western Blot, respectively. Finally, to explore whether miR-3940-5p affects Aβ production, SH-SY5Y APP^swe^ cells overexpressing the Swedish mutant type of APP were transiently transfected with miR-3940-5p mimics, and the expression level of Aβ was detected using ELISA. The results are as follows: The dual-luciferase reporter assays validated the targeting specificity of miR-3940-5p for *PSEN1*. Overexpression of miR-3940-5p significantly reduced the mRNA and protein levels of *PSEN1* in SH-SY5Y cells. Conversely, inhibition of miR-3940-5p led to an increase in *PSEN1* mRNA levels. Transfection of miR-3940-5p mimics into SH-SY5Y-APP^swe^ cells resulted in a significant reduction in Aβ_42_ and Aβ_40_. Lentiviral-mediated overexpression of miR-3940-5p significantly decreased the expression of *PSEN1* and did not significantly affect the expression of other predicted target genes. Furthermore, stable overexpression of miR-3940-5p in SH-SY5Y-APP^swe^ cells mediated by lentivirus significantly reduced the expression of *PSEN1* and the production of Aβ_42_ and Aβ_40_. Therefore, our study demonstrates for the first time the functional importance of miR-3940-5p in antagonizing Aβ production through specific and direct targeting of *PSEN1*.

## 1 Introduction

Alzheimer disease (AD) is a progressive neurodegenerative disorder manifested as cognitive, mood, language, and memory impairments. As the primary cause of dementia in the elderly, AD has multiple contributing factors, with Aβ being widely acknowledged as one of the foremost factors influencing the onset and progression of AD (Hardy and Selkoe, 2002). Aβ is a series of peptide fragments formed after the cleavage of amyloid precursor protein (APP) by β- and γ-secretase enzymes. The excessive aggregation of Aβ into amyloid plaques disrupt the structural integrity of dendritic spines and synapses, resulting in a cascade of deleterious effects that ultimately lead to neuronal death (Ingelsson et al., 2004). Additionally, Aβ oligomers have the potential to inflict direct damage upon the synapses and neurites of brain neurons, along with the activation of microglia and astrocytes (Selkoe and Hardy, 2016). Clinical trials utilizing monoclonal antibody (Donanemab) to selectively remove Aβ have demonstrated significant reductions in cognitive and functional decline in patients of early-stage AD (Mintun et al., 2021). Crucially, recent clinical trials involving three distinct Aβ antibodies—solanezumab, crenezumab, and aducanumab—indicate a potential deceleration in cognitive decline, as observed in *post hoc* analyses of individuals with mild AD (Selkoe and Hardy, 2016), confirming the critical role of Aβ in AD pathogenesis. Therefore, identifying molecular targets that specifically inhibit or modulate Aβ production has become a research focus in AD.

Aβ is produced through the amyloidogenic pathway during the degradation of APP, where β-secretase (BACE1) cleaves APP to produce β-CTF, which is further cleaved by γ-secretase to generate Aβ_48_/Aβ_49_ and APP intracellular domain. Finally, γ-secretase trims Aβ_48_/Aβ_49_ to produce Aβ of varing lengths (38–43 amino acids), with Aβ_40_ accounting for ~90% of Aβ production (Bolduc et al., 2014). APP can also be processed by a non-amyloidogenic pathway mediated by α-secretase (ADAM10) and γ-secretase, thereby suppressing the production of Aβ. Unlike the α- and β-secretases that are single-protein enzymes, γ-secretase is a multi-subunit enzyme composing by at least four subunits—namely, presenilin (PSEN), nicastrin (NCT), anterior pharynx defective 1 (APH1) and presenilin enhancer 2 (PEN2) (Jayne et al., 2016). PSEN constitutes the catalytic core of γ-secretase and, PSEN1 and PSEN2 are two members of mammalian PSEN family. Although both PSEN1 can be incorporated into γ-secretase, PSEN1 has gained more research interests because over 80% of mutations linked to familial AD locate in *PSEN1* (Zoltowska and Berezovska, 2018).

miRNAs are endogenous, non-coding RNAs ranging from 19 to 24 nucleotides in length, and present in various eukaryotic organisms. miRNAs regulate gene expression by specifically targeting the 3′ untranslated region (3’UTR) of mRNA. miRNAs dysfunction is associated with a myriad of human diseases and is a target for disease diagnose and therapy. Recent studies have demonstrated that the dysregulation of miRNA expression has become a crucial factor in AD pathology (Fehlmann et al., 2020; Sproviero et al., 2021). Various miRNAs are involved in regulating Aβ, tau, and neuroinflammation. To identify miRNAs that have specific role in regulating Aβ production, most studies focused on miRNAs targeting *APP* (Patel et al., 2008; Hébert et al., 2009; Vilardo et al., 2010; Liang et al., 2012), *BACE1* (Cheng et al., 2014; Deng et al., 2014; Xie et al., 2017; Chopra et al., 2021) and *ADAM10* (Cheng et al., 2013; Sun et al., 2017; Akhter et al., 2018; Lu et al., 2019; Sarkar et al., 2019). miRNAs targeting *PSEN1*, which encode the catalytic subunit of γ-secretase, is extremely understudied. Currently, only miR-29b-2-5p (Wuli et al., 2022) and miR-647 (Li et al., 2022) have been reported to target *PSEN1*.

Since γ-secretase is a rate-limiting enzyme in Aβ biogenesis, studying how PSEN1 is regulated by miRNAs may provide a promising therapeutic approach for AD. To this end, this study aimed to investigate the regulatory role of miRNAs on the expression of *PSEN1*. We utilized bioinformatics tools such as Target Scan and experimental techniques including dual-luciferase reporter analysis, transient and stable transfection, RT-qPCR, western blot, and ELISA to identify candidate miRNAs that potentially target *PSEN1*. We confirmed that miR-3940-5p directly targets the 3’UTR of *PSEN1* and reduces its translation level in SH-SY5Y cells. Upon miR-3940-5p overexpression, the generation of Aβ_42_ and Aβ_40_ in SH-SY5Y-APP^swe^ cells was significantly decreased. Our study highlights the regulatory role of miRNAs in AD pathogenesis and identifies miR-3940-5p as a potential therapeutic target for AD.

## 2 Materials and methods

### 2.1 Cell culture

In this study, we used four cell lines, namely HEK 293, HEK 293 T, SH-SY5Y, and SH-SY5Y-APP^swe^. SH-SY5Y-APP^swe^ was genetically modified to overexpress *APP^swe^* in SH-SY5Y. These cells were obtained from Kunming Institute of Zoology of the Chinese Academy of Sciences (HEK 293, HEK 293 T and SH-SY5Y) or Kunming Medical University (SH-SY5Y-APP^swe^), were cultured in DMEM supplemented with 10% fetal bovine serum (Gbico, Waltham, MA, USA). Prior to experimentation, the cells were harvested using trypsinization and counted using the trypan blue exclusion method when they reached approximately 70% confluency. For the experiments, 150,000 cells were seeded into each well of a 24-well plate (Corning, New York, USA), while 50,000 cells were seeded into each well of a 96-well plate (Corning, New York, USA).

### 2.2 Target prediction

To predict miRNAs targeting the PS1 3’UTR, this study employed three prediction websites: TargetScan (Agarwal et al., 2015), miRDB (Wong and Wang, 2015), and miRwalk (Dweep et al., 2011). TargetScan, a miRNA target gene prediction database, yielded results for various mammalian miRNA target genes. It relies on the principle of sequence complementarity to identify conservative 8-mer, 7-mer, or 6-mer sites (seed match sequences) within the target gene’s 3’UTR. Further refinement, based on thermodynamic stability, was applied to select miRNAs targeting the gene of interest, with a preference for those with a context score percentile exceeding 90 for subsequent validation. miRDB, a database for predicting mammalian miRNA target genes, assigned higher credibility to results with scores above 80, considered relatively reliable. miRWalk, a comprehensive miRNA target gene database, encompassed information for multiple species, including Human, Mouse, Rat, Dog, Cow, etc. It employed four different algorithms (RNA22, miRanda, miRWalk, and TargetScan) to predict gene-targeting miRNAs and integrated this information with predictions from 12 existing miRNA target prediction programs. To reduce false positives, predictions from each of the three databases regarding miRNAs targeting the PS1 3’UTR were consolidated. The Whitehead BaRC public tools website was then used to obtain their intersection. Ultimately, five miRNAs with high scores and no prior literature reports were selected for further validation: miR-9-5p, miR-302a-3p, miR-520c-3p, miR-4507-5p, and miR-3940-5p.

### 2.3 Dual luciferase report analysis

HEK 293 cells were seeded onto a 96-well plate at a density of approximately 50,000 cells per well, 1 day prior to transfection. For the dual-luciferase reporter assay, 800 ng of the psiCHECK2 plasmid containing either wild-type or mutated *PSEN1* 3′UTR was independently co-transfected with 100 nM miR-3940-5p mimics (Ribobio, Guangzhou, China) using Lipofectamine 2000 (Invitrogen, Waltham, MA, USA). After 48 h of incubation, the cells were lysed and subjected to the reporter assay (Promega, Wisconsin, USA).

### 2.4 Transient transfection

For SH-SY5Y cells transfections, ~150,000 cells/well were seeded onto a 24-well plate. The following day, Lipofectamine 2000 (5 μL) were made with 100 nM miRNA mimics, inhibitor, negative control or 100 nM siRNA (*PSEN1* siRNA, general biol, Anhui, China) (Guo et al., 2023) in Opti-MEM media (Gbico, Waltham, MA, USA) complexes were allowed to form for 20 min before being added to cells. Then 500 μL of media (no antibiotics) was added to each well. Cells were incubated 48 h (RT-qPCR) or 72 h (western blot) until lysis.

### 2.5 Lentivirus packaging and stable cell line generation

To package the slow virus, the virus genome is cloned into a specialized vector containing all the essential elements for viral replication and packaging. Specifically, a chemical synthesis method is used to clone the fragment of the precursor structure of miR-3940-5p (miR-3940-5p-pre), which encodes it, into the slow virus vector (pCDH-CMV-MCS-EF1-copGFP-T2A-Puro vector). The precursor sequence that encodes miR-3940-5p (miR-3940-5p-pre): 5’TCTAGACGAATAGCTCCTTTTCTAGCTCCACCCAACCCCGC CCGAGACCCCTAAACCAGAGTGTCGGGCCTAGGGTCGGGTG AATGGAACCAATGAGAGGAAGGAAGATTTTTG GATCC3’. The vector is then transfected into a packaging cell line that contains a helper virus. The helper virus provides the proteins and enzymes required for the slow virus to replicate and package its genome. The slow virus genome is integrated into the host cell genome through a process known as transduction. Subsequently, the packaging cell line is infected with the helper virus, which initiates the production of slow virus particles. The slow virus particles are then released from the packaging cell line and can infect target cells.

To overexpress miR-3940-5p, SH-SY5Y cells were seeded in triplicate in a six-well plate in the following order: blank group, overexpressing miR-3940-5p group, and negative control group. When the cells reached about 70% confluency, a mixture of complete culture medium, corresponding virus solution, and polybrene (10 mg/mL mother liquor, 10 μg/mL final concentration) (Beyotime, Shanghai, China) was added to the six-well plate, replacing the original culture medium. The volume of each component was 1 mL, 1 mL, and 2 μL, respectively. Normal culture medium was substituted 24 h later. After 48 h, the culture medium was replaced with medium containing puromycin (Beyotime, Shanghai, China), and the concentration was adjusted based on the screening outcomes (Final concentration of 1 μg/mL). Following 3–4 days of puromycin drug treatment, wild-type SH-SY5Y cells perished, and cells stably overexpressing miR-3940-5p were obtained and denoted as SH-SY5Y-miR-3940-5p-pre cells. We used the same procedures to obtain SH-SY5Y-APP^swe^ cells stably overexpressing miR-3940-5p, which were denoted as SH-SY5Y-APP^swe^-miR-3940-5p-pre cells.

### 2.6 The reverse transcription real-time quantitative PCR (RT-qPCR)

RNA was extracted from SH-SY5Y cells using a modified protocol from the miRNA Isolation kit (OMEGA-Bio-Tek, Texas, USA). The extracted RNA was eluted in 50 μL of nuclease-free water and quantified to be used as a template for cDNA synthesis (TIANGEN, Beijing, China and Takara, Kyoto, Japan). SH-SY5Y cells were transfected with miR-3940-5p mimics and inhibitor, and the expression levels of *miR-3940-5p* and *PSEN1* were analyzed by RT-qPCR (TIANGEN, Beijing, China and KAPA, Boston, USA) 48 h after transfection. Primer sequence of *miR-3940-5p*: 5’GUGGGUUGGGGCGGGCUCUG3’, *PSEN1* F: 5’ACAGGTGCTAT AAGGTCATCCA3’, *PSEN1* R: 5’CAGATCAGGAGTGCAACAGTA AT3’, *GAPDH* F: 5’AGATCATCAGCAATGCCTCCT3’, *GAPDH* R: 5’GGTCATGAGTCCTTCCACGA3’. Ct values were determined using a constant threshold, and fold change was calculated by the delta–delta Ct method.

### 2.7 Western blotting

After transfection miR-3940-5p mimic for 5 h, the cells were exchanged with normal culture medium and incubated for an additional 72 h. In addition, during the logarithmic growth phase, total protein was extracted from SH-SY5Y cell lines overexpressing miR-3940-5p, western blot technique can be used to detect protein expression levels, which involves protein extraction, protein concentration measurement, protein electrophoresis, protein transfer, blocking, primary antibody incubation (Anti-PSEN1, 1: 6000, Abcam, Cambridge, UK, Anti-GAPDH, 1: 2000, Beyotime, Shanghai, China), washing, secondary antibody (Abcam, Cambridge, UK) incubation, detection, and data analysis using software such as Image J.

### 2.8 Enzyme labeled immunosorbent assay (ELISA)

The levels of both soluble peptides were measured in the conditioned media of transfected cells using ELISA kits (Jiangsu Enzyme Label Biotechnology, Jiangsu, China) for Aβ_40_ and Aβ_42_. A standard curve was generated according to the protocol to obtain the concentration of each Aβ peptide (pg/μg). In this context, “pg” represents the amount of Aβ measured in each well, while “μg” represents the total protein content in each well.

### 2.9 Statistical approach

The data are presented as mean ± standard error. The comparison of means between two groups was performed using a *t*-test. Statistical analysis was conducted using SPSS 20.0 software, and graphical representations were created using GraphPad Prism 5.

## 3 Results

### 3.1 miR-3940-5p targets the *PSEN1* 3’UTR by double luciferase report analysis

**Figure 1:**
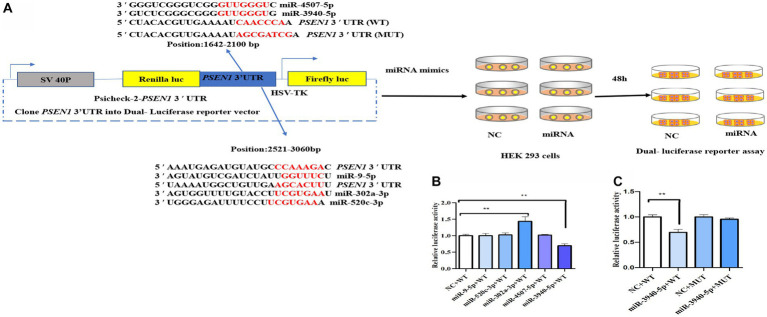
miR-3940-5p specifically targets the 3′UTR of *PSEN1*. **(A)** Schematic representation of the experimental design. The wild-type (WT) *PSEN1* 3′UTR was cloned downstream to the renilla luciferase gene in psiCheck 2 vector to generate the reporter constructs. The HEK293 cells were transfected with the luciferase reporter constructs and with candidate miRNA (miR-9-5p, miR-302a-3p, miR-520c-3p, miR-3940-5p, or miR-4507-5p) or negative controls (NC). After 48 h, dual luciferase assay **(B)** was performed. As shown in **(A)**, the *PSEN1* 3′UTR with mutated miR-3940-5p target site (MUT) was constructed into psiCheck 2 vector. Then, HEK293 cells were transfected with constructs containing WT or MUT 3′UTR and miR-3940-5p or NC. After 48 h, dual luciferase assay **(C)** was performed (*n* = 3, *t* test, data represent mean ± SEM., **p < 0.01).

To screen potential miRNAs targeting the *PSEN1* 3’UTR, target prediction websites including Target Scan, miRDB, and miRWalk were utilized. The identified miRNA candidates were further refined by utilizing the Whitehead BaRC public tools website to determine their intersection. Figure 1A depicts the experimental design of the dual-luciferase reporter assay. The wild-type (WT) PSEN1 3’UTR was cloned downstream of the renilla luciferase gene in the psiCheck 2 vector to generate the reporter constructs. HEK293 cells were transfected with the luciferase reporter constructs along with candidate miRNA (miR-9-5p, miR-302a-3p, miR-520c-3p, miR-3940-5p, or miR-4507-5p) or negative controls (NC). After 48 hours, dual-luciferase assays were performed. As a result, miR-9-5p, miR-302a-3p, miR-520c-3p, miR-3940-5p, and miR-4507-5p were selected. To test whether miR-9-5p, miR-302a-3p, miR-520c-3p, miR-3940-5p, and miR-4507-5p could bind to the 3’UTR of *PSEN1*, the luciferase activity of HEK293 cells was measured. The candidate miRNA mimics (100 nM) and wild-type plasmids (0.8 μg) were co-transfected, and the relative activity of firefly luciferase was determined. The results (Figure 1B) showed that only the miR-3940-5p mimic could significantly reduce the activity of the dual-luciferase system compared to the control (*p* < 0.01). The miR-9-5p, miR-302a-3p, miR-520c-3p, and miR-4507-5p mimics did not significantly change the activity of the dual-luciferase system, and the miR-302a-3p group showed a significant increase (*p* < 0.01). These data indicated *PSEN1* is the target of miR-3940-5p.

To verify this, the seed sequence of miR-3940-5p in the *PSEN1* 3’UTR was mutated and constructed into the reporter vector. No changes in luciferase activity were seen when the constructs contained a mutated miR-4507-5p seed region (Figure 1C), indicating that the targeting effect of miR-3940-5p on the mutated *PSEN1* was lost. Overall, these findings confirmed that miR-3940-5p directly targets *PSEN1*.

### 3.2 miR-3940-5p inhibits the PSEN1 transcription and translation in SH-SY5Y cells

**Figure 2:**
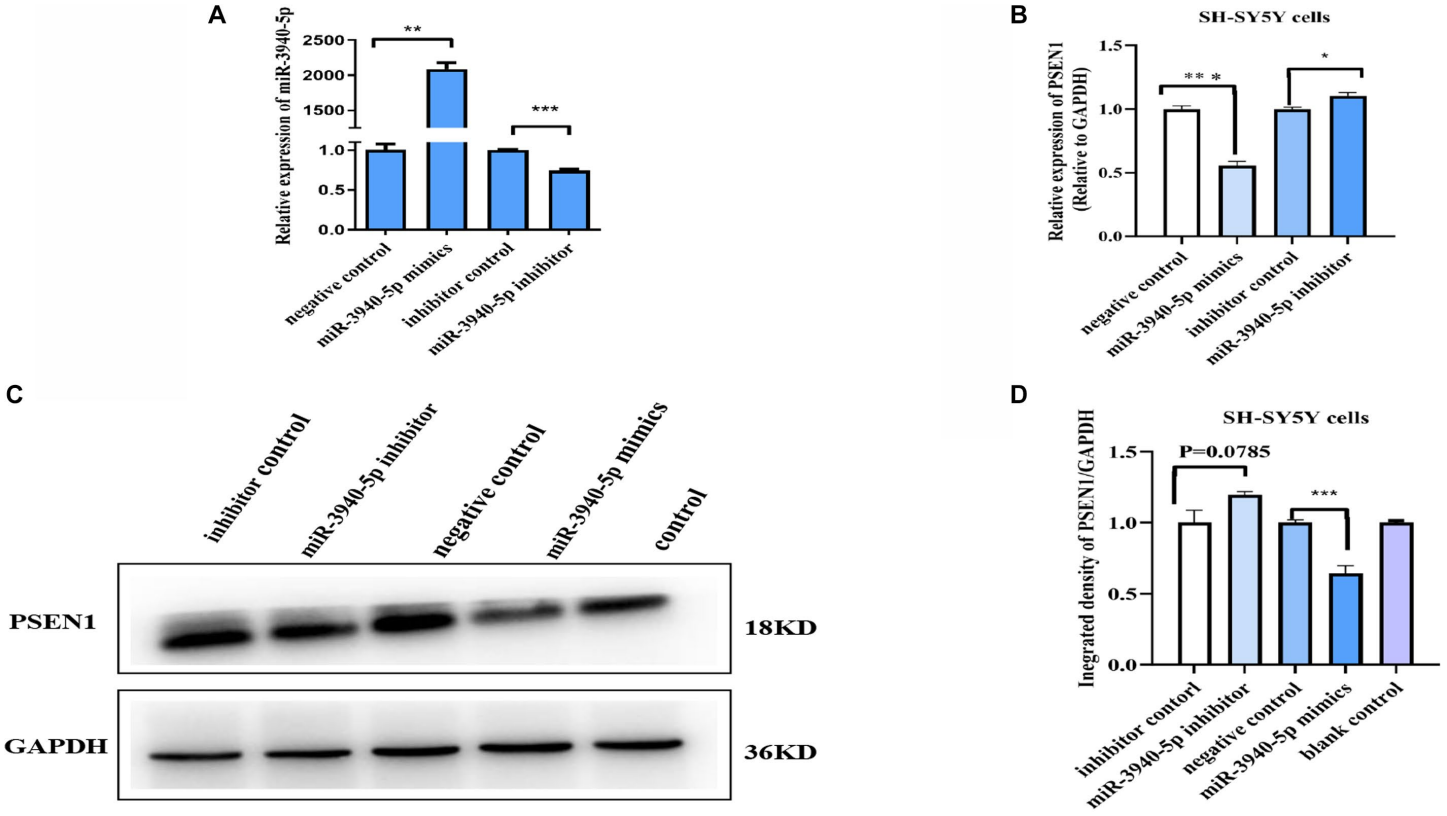
miR-3940-5p inhibits the transcription and translation of *PSEN1* in SH-SY5Y cells. **(A)** Transient transfection of miR-3940-5p mimics or inhibitors to SH-SY5Y cells results in miR-3940-5p overexpression or suppression. **(B)** miR-3940-5p mimics significantly reduce the mRNA level of *PSEN1* and miR-3940-5p inhibitor increase the mRNA level of *PSEN1*. **(C, D)** Transfection with a miR-3940-5p mimic resulted in a significant decrease in PSEN1 protein levels, while addition of a miR-3940-5p inhibitor increased *PSEN1* protein levels (*n* = 3, *t* test, data represent mean ± SEM., **p < 0.01, ***p < 0.001).

To investigate the role of miR-3940-5p on PSEN1 further, we used the chemically synthesized miR-3940-5p mimics and inhibitors to overexpress and down express miR-3940-5p in SH-SY5Y cells. 48 h after miR-3940-5p mimics transfection, the RT-qPCR analysis (Figure 2A) revealed that the expression of miR-3940-5p is increased by approximately 2000 times compared to the control group, indicating the successful overexpression of miR-3940-5p in the cells. Conversely, 48 h after miR-3940-5p inhibitor transfection, the expression of miR-3940-5p is decreased by 30% (Figure 2A). These data confirmed that miR-3940-5p mimics and inhibitors are functional in SH-SY5Y cells.

Next, miR-3940-5p mimics and inhibitors were transfected into SH-SY5Y cells and the expression levels of *PSEN1* mRNA and protein were analyzed. RT-qPCR results (Figure 2B) revealed a significant decrease in *PSEN1* mRNA levels in the miR-3940-5p group compared to the control group (*p* < 0.01). Western blot results (Figures 2C, D) showed that PSEN1 protein expression was significantly inhibited in the miR-3940-5p mimics group compared to the control group, which was consistent with the observed decrease in *PSEN1* mRNA expression. When miR-3940-5p inhibitor was transfected into SH-SY5Y cells, the mRNA (*p* < 0.05; Figure 2B) and protein (*p* = 0.051; Figure 2C) levels of PSEN1 were significantly increase, although the increase in protein was marginally significant. Altogether, these results further confirm the negative regulation of *PSEN1* expression by miR-3940-5p in SH-SY5Y cells.

### 3.3 PSEN1 is down-expressed in cells stably overexpressing miR-3940-5p

To further investigate the regulatory role of miR-3940-5p on PSEN1, we constructed a lentiviral vector by inserting the 119 bp sequence encoding the precursor structure of miR-3940-5p. This was achieved through xbaI/BamH I double enzyme digestion. The lentiviral plasmid carrying the correct miR-3940-5p sequence was then packaged into lentivirus and used to overexpress miR-3940-5p in SH-SY5Y cells. In addition to the miR-3940-5p sequence, the lentiviral vector also contained a green fluorescent protein gene (*GFP*). These features allowed us to select miR-3940-5p-overexpressing SH-SY5Y cells using puromycin and visualize them under a fluorescence microscope.

**Figure 3:**
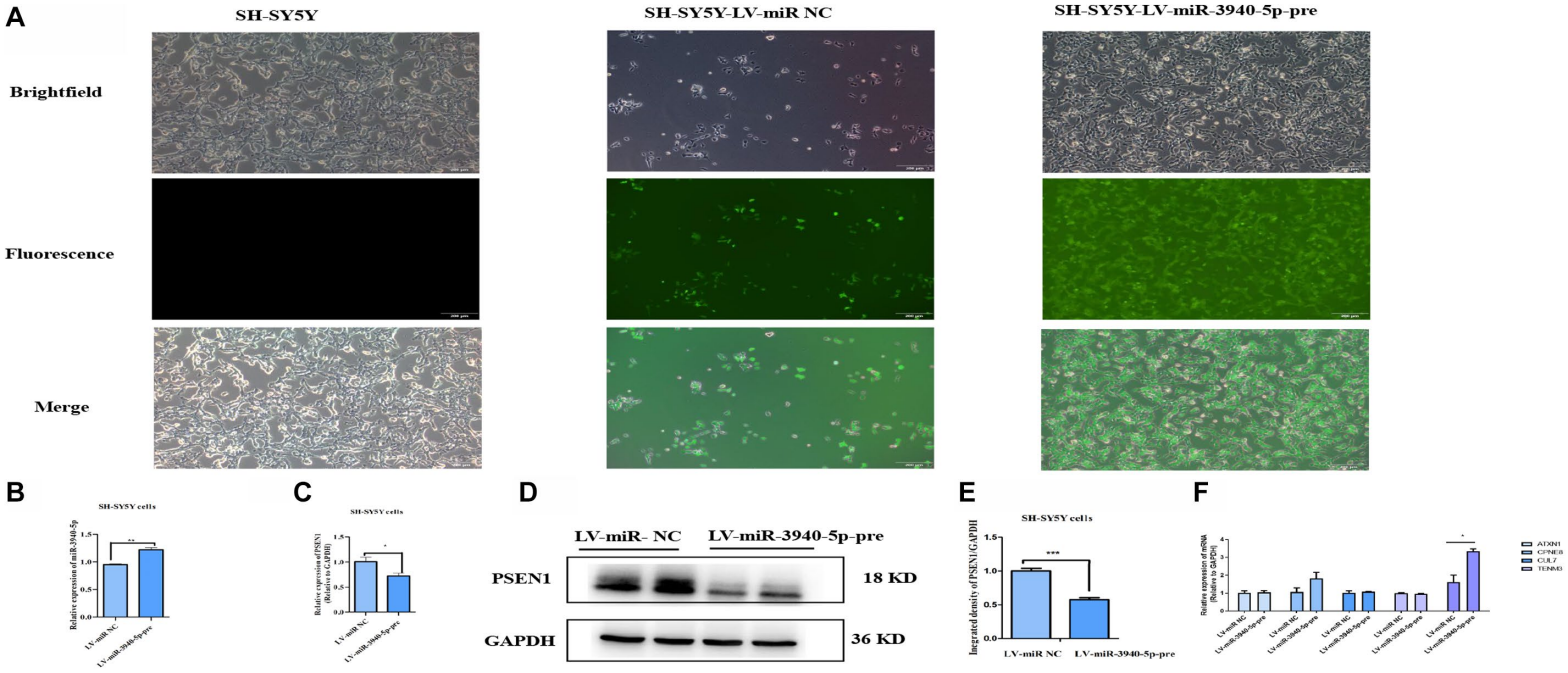
PSEN1 is down-expressed in cells stable-overexpressing miR-3940-5p. **(A)** After puromycin selection, the morphology of SH-SY5Y cells overexpressing miR-3940-5p were captured under brightfield and fluorescence microscopy. **(B)** The expression of miR-3940-5p was detected in miR-3940-5p overexpressing cells and control cells through RT-qPCR. **(C)** The expression of *PSEN1* in overexpressing cells was quantified using RT-qPCR. **(D, E)** The expression level of PSEN1 was detected in miR-3940-5p overexpressing cells through western blot. **(F)** The expression of *TENM3*, *CPNE8*, *ATXN1*, *CUL7*, and *KCNA5* was quantified using RT-qPCR in SH-SY5Y cells overexpressing miR-3940-5p (*n* = 3, *t* test, data represent mean ± SEM., *p < 0.05,**p < 0.01, ***p < 0.001).

Following puromycin selection, SH-SY5Y cells with miR-3940-5p overexpression (termed as SH-SY5Y-miR-3940-5p-pre) displayed green fluorescence when observed under a fluorescence microscope (Figure 3A). We detected the expression level of miR-3940-5p in SH-SY5Y-miR-3940-5p-pre cells using RT-qPCR. Compared to the LV-miR-NC group, the expression of miR-3940-5p in the LV-miR-3940-5p-pre group was increased by 20%, confirming a successful overexpression of miR-3940-5p (Figure 3B). The level of miR-3940-5p in SH-SY5Y-miR-3940-5p-pre cells was not very high, probably due to the complexity of the transcriptional regulation of miR-3940-5p precursor that ultimately leads to the formation of both mature miR-3940-5p and miR-3940-3p.

Subsequently, both RNA and protein were extracted from SH-SY5Y-miR-3940-5p-pre cells. The mRNA level of *PSEN1* was measured using RT-qPCR, and the results indicated that the overexpression of miR-3940-5p considerably reduced the expression of *PSEN1* (Figure 3C). In addition, the protein level of PSEN1 was analyzed through western blot, and the data showed that the overexpression of miR-3940-5p significantly reduced PSEN1 expression (Figures 3D, E). These results demonstrated that the mRNA and protein expression of PSEN1 were significantly declined in SH-SY5Y-miR-3940-5p-pre cells.

To validate the specificity of miR-3940-5p targeting *PSEN1* in SH-SY5Y cells, we employed miRNA target prediction websites, including Target Scan, miRDB, and miRwalk, to screen for the potential miR-3940-5p targets. The top five genes targeted by miR-3940-5p with high prediction scores were *TENM3*, *CPNE8*, *ATXN1*, *CUL7*, and *KCNA5.* RT-qPCR was carried out to assess the expression levels of these five genes in SH-SY5Y-miR-3940-5p-pre cells. The results (Figure 3F) showed that, compared to the control group, the expression level of *KCNA5* was significantly upregulated in SH-SY5Y-APP-miR-3940-5p cells, while the expression levels of other three genes remained unchanged (Figure 3F). Thus, miR-3940-5p does not target these 5 genes, to some extent, had specificity toward *PSEN1*.

### 3.4 miR-3940-5p inhibits Aβ production In SH-SY5Y-APP^swe^ cells

**Figure 4:**
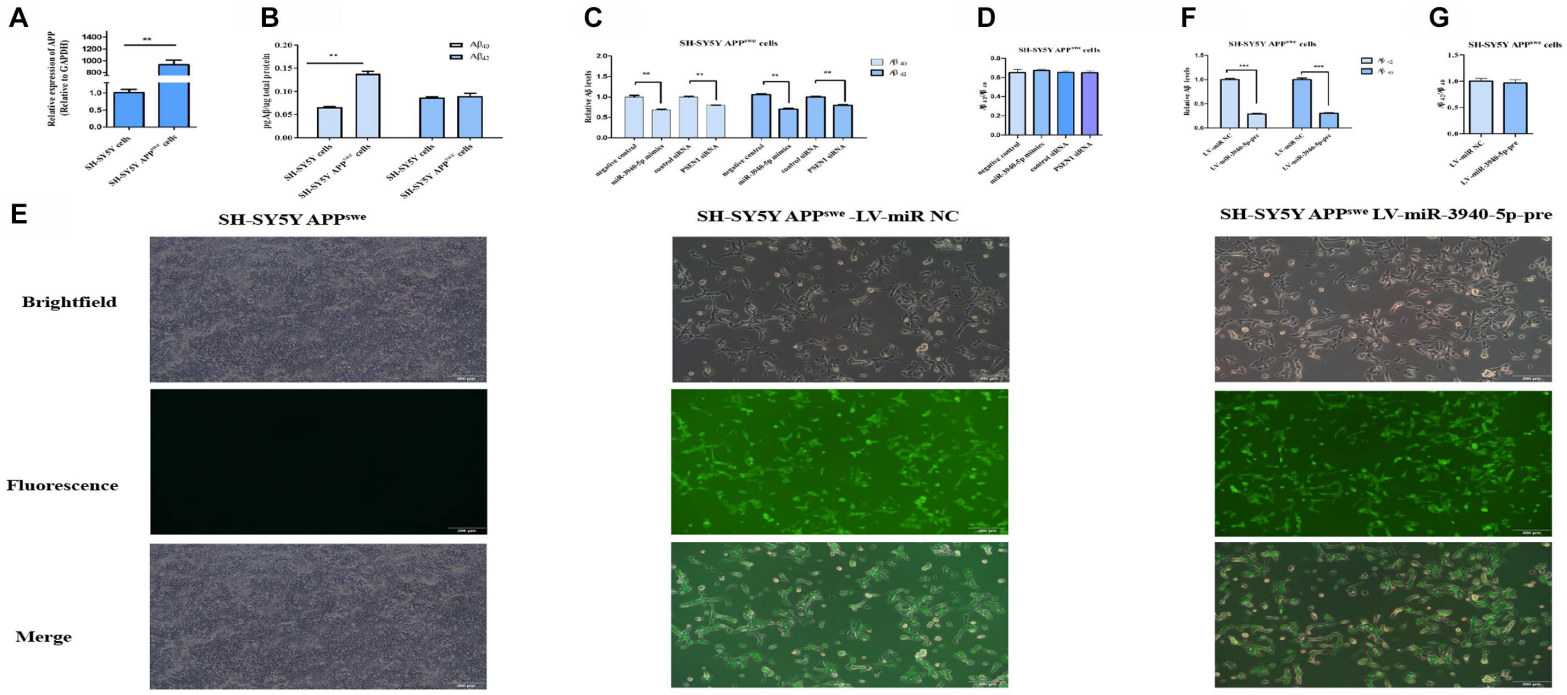
miR-3940-5p inhibits the production of Aβ in SH-SY5Y-APP^swe^ cells. **(A)** RT-qPCR result shows that the *APP* is overexpressed in SY5Y-APP^swe^ cells. **(B)** A comparison of the Aβ_40_ and Aβ_42_ concentrations in SH-SY5Y-APP^swe^ cells and SH-SY5Y cells. **(C)** miR-3940-5p and PSEN1 siRNA reduce the production of Aβ in SH-SY5Y-APP^swe^ cells. **(D)** The ratio of Aβ_42_ to Aβ_40_ in cells treated by miR-3940-5p mimics and PSEN1 siRNA. **(E)** After puromycin selection, the morphology images of SH-SY5Y-APP^swe^ cells overexpressing miR-3940-5p were captured under brightfield and fluorescence. After overexpression of miR-3940-5p in SH-SY5Y-APP^swe^ cells, the production of Aβ_40_ and Aβ_42_ **(F)**, and the ratio of Aβ_42_ to Aβ_40_ **(G)** were measured in this cell line (*n* = 3, *t* test, data represent mean ± SEM., ***p < 0.001).

PSEN1 is a crucial component of the γ-secretase complex, which plays a rate-limiting role in regulating Aβ production. Considering this, inhibiting *PSEN1* expression may represent a potential therapeutic strategy for AD by reducing brain Aβ levels. To explore the impact of miR-3940-5p on Aβ production, SH-SY5Y-APP^swe^ cells were utilized as a research model. This cell line is an AD cell model that can produce neurotoxic Aβ. RT-qPCR (Figure 4A) was employed to evaluate the expression of *APP* in this cell line and we found the expression of *APP* in SH-SY5Y-APP^swe^ cells was 120 times higher than that in SH-SY5Y cells. Moreover, ELISA was used to quantify the expression level of Aβ in both SH-SY5Y and SH-SY5Y-APP^swe^ cells. The data (Figure 4B) depicts the results of Aβ_40_ and Aβ_42_ content analysis in SH-SY5Y and SH-SY5Y-APP^swe^ cells, which demonstrates that the Aβ_40_ content in SH-SY5Y-APP^swe^ cells is significantly higher compared to SH-SY5Y cells. Although the Aβ_42_ content is also higher in SH-SY5Y-APP^swe^ cells, the difference is not statistically significant. These results confirmed that SH-SY5Y-APP^swe^ cells have a high expression of APP and Aβ.

To explore the downstream impact of miR-3940-5p on Aβ production, miR-3940-5p mimics were transiently transfected into SH-SY5Y-APP^swe^ cells and ELISA was utilized to assess the expression levels of Aβ_40_ and Aβ_42_ thereafter. Meanwhile, a specific small interference RNA to PSEN1 (Guo et al., 2023) was used as a control. The results (Figure 4C) revealed that both the miR-3940-5p mimics and *PSEN1*-siRNA could significantly suppress the production of Aβ_40_ and Aβ_42_ in SH-SY5-APP^swe^ cells. Since the increase in the relative ratio of Aβ_42_ to Aβ_40_ (Aβ_42_/Aβ_40_) is a common result of familial AD-associated PSEN1 mutations (Sun et al., 2017) and plays an important role in AD pathogenesis (Kwak et al., 2020), we determined the effect of miR-3940-5p on the ratio of Aβ_42_/Aβ_40._ The data showed that there is no significant difference in the ratio of Aβ_42_/Aβ_40_ cross the miR-3940-5p mimics group, *PSEN1*-siRNA group, and the control group (Figure 4D).

Next, the lentiviruses system constructed above was used to stably overexpress miR-3940-5p in SH-SY5Y-APP^swe^ cells. The success of miR-3940-5p overexpression was confirmed through fluorescence microscopy. The levels of Aβ_40_ and Aβ_42_ in SH-SY5Y- APP^swe^ cells stably expressing miR-3940-5p-pre were analyzed using ELISA. The results (Figure 4E) showed a significant reduction in the levels of Aβ_40_ and Aβ_42_ in SH-SY5Y- APP^swe^-miR-3940-5p-pre cells compared to the control group. Regarding the ratio of Aβ_42_/Aβ_40_, there were no significant differences observed between the SH-SY5Y-APP^swe^-miR-3940-5p group and the control group (Figure 4F). These findings were consistent with the results obtained from transient miR-3940-5p mimics transfection.

## 4 Discussion

The pathogenesis of AD is multifaceted and not yet fully understood. Mounting evidence has highlighted the crucial role of miRNAs in regulating gene expression in the brain, where they constitute a significant regulatory system (Dehghani et al., 2018). Several studies have demonstrated that miRNAs regulate Aβ generation by targeting BACE1. For instance, miR-29c-3p (Cao et al., 2021), miR-195 (Zhu et al., 2012), miR-186 (Kim et al., 2016), miR-200a-3p (Wang et al., 2019). While miRNAs have been shown to play a crucial role in regulating β-secretase, miRNAs targeting γ-secretase are rarely reported. In current study, we provided sold evidence that miR-3940-5p is one miRNA specifically target *PSEN1* and regulate the γ-secretase function of PSEN1.

We initially used bioinformatics screening to discover the potential miRNAs with high mRNA: miRNA binding affinity toward *PSEN1*. With five candidate miRNAs in hand, we used various experimental approaches to validate the computational predictions and demonstrate that the miR-3940-5p recognize specific binding sites in the 3’UTR of *PSEN1* mRNA and suppress PSEN1 protein expression in SH-SY5Y cells.

Lentiviral-mediated overexpression of miRNAs is a principal method for studying miRNA functions as it has lower cell toxicity and allows for stable overexpression of the target miRNA in a short period of time (Herrera-Carrillo et al., 2009). Compared with the control cell line, the expression level of miR-3940-5p in the LV-miR-3940-5p-pre group was increased by 20%. The mild increase of miR-3940-5p in SH-SY5Y-miR-3940-5p cells is possibly due to the complexity of the miRNA transcription process when the exogenous encoding miR-3940-5p precursor sequence is inserted into the genome DNA, resulting in the formation of two mature miRNAs, miR-3940-5p and miR-3940-3p (O'Brien et al., 2021). Despite miR-3940-5p was mildly increased, the mRNA and protein levels of *PSEN1* were significantly reduced, indicating the 20% increase of miR-3940-5p in SH-SY5Y-miR-3940-5p cells was functional.

In present study, transient and stable transfection methods were used to investigate the regulatory effects of miR-3940-5p on the production of Aβ Transfection of miR-3940-5p mimics and *PSEN1*- siRNA into SH-SY5Y-APP^swe^ cells resulted in a decrease in both Aβ_40_ and Aβ_42_ levels. Notably, the stable overexpression of miR-3940-5p in SH-SY5Y-APP^swe^ cells led to a decrease in both Aβ_40_ and Aβ_42_ levels, without altering the Aβ_42_/Aβ_40_ ratio, which is consistent with the results of transient transfection. Notably, the stable overexpression of miR-3940-5p in SH-SY5Y-APP^swe^ cells led to a decrease in both Aβ_40_ and Aβ_42_ levels, without altering the Aβ_42_/Aβ_40_ ratio, which is consistent with the results of transient transfection. These findings demonstrate that, by suppressing the expression of PSEN1 and the consequent γ-secretase activity, miR-3940-5p is involved in reducing the production of Aβ. In future studies, it would be worthwhile to overexpress PSEN1 in SH-SY5Y cells and transfect them with miR-3940-5p to explore whether overexpression of PSEN1 can rescue the inhibitory effect of miR-3940-5p on Aβ levels.

RNA-targeted therapy, which regulates protein synthesis by designing antisense oligonucleotides (ASOs), is a promising approach for treating AD. ASOs based on miRNAs, including antagomiRs and miRNA mimics, have been shown to be potential targets. Several miRNAs based ASOs drugs have been tested in preclinical studies and demonstrated promising results (Grabowska-Pyrzewicz et al., 2021). By employing several *in vitro* functional experiments, we have shown that miR-3940-5p indirectly reduces the generation of Aβ by targeting *PSEN1*, providing a foundation for future *in vivo* experiments. Interestingly, one study has revealed that, by injecting a lentivirus-packaged miR-31 overexpression plasmid into the hippocampus of AD mice, the expression of App and Bace1 are declined, which finally reduces Aβ deposition and improves cognitive ability (Barros-Viegas et al., 2020). This study has inspired us to test whether overexpressing miR-3940-5p in AD mice would exert similar benefits. However, among the organisms present in the TargetScan database, including mouse, chimpanzee, and other nine species, no homologous miRNA for miR-3940-5p has been identified. This indicates that miR-3940-5p is evolutionarily non-conserved and is a human-specific miRNA. To substantiate the presumed human specificity of miR-3940-5p and its potential association with the onset and progression of Alzheimer’s disease, a more rigorous inquiry is required. In subsequent investigations, we plan to meticulously compare and analyze the genomes of humans and other pertinent species (such as chimpanzees, mice, etc.) to conclusively establish whether this miRNA is uniquely present in humans. Following this, we will employ high-throughput sequencing technology to conduct an extensive miRNA expression profiling, comparing the expression patterns of miR-3940-5p between humans and other species.

Besides, *in vivo* validating the function of miR-3940-5p in current-available mouse model is challenging. The seed sequence of miR-3940-5p targeting to *PSEN1* locates on the 3’UTR. In most transgenic AD mice based on *PSEN1*, like APP/PSEN1 mice, only the coding sequence of mutated *PSEN1* is transferred. Therefore, the human derived 3D brain organoids may be an ideal model to address this issue. Considering that the PSEN1 in brains of sporadic AD is upregulated (Borghi et al., 2010), it is worth to investigate whether miR-3940-5p in AD brains or serum is correspondingly downregulated. Upon confirmation of the human specificity of miR-3940-5p, we intend to delve into its origin and evolutionary trajectory in the human lineage. This endeavor is poised to enhance our comprehension of genetic disparities between humans and other species, shedding light on how these distinctions may impact biological functionalities and adaptability.

Previously, miR-3940-5p has found to target several genes. However, most of them were validated in cancer. For example, miR-3940-5p suppresses colorectal cancer metastasis by targeting integrin alpha 6 (Tao et al., 2021). miR-3940-5p suppresses the proliferation of non-small cell lung cancer cells by targeting cyclin D1 and ubiquitin specific peptidase-28 (Ren et al., 2017). In addition, miR-3940-5p is a hub miRNA upregulated in granulosa cells from patients with polycystic ovary syndrome and promotes granulosa cell proliferation by targeting *KCNA5* (Gao et al., 2020). Coupled with our present study, these findings demonstrate that miR-3940-5p has a pleiotropic role in human health. However, the therapeutic use of miRNAs with a multitude of targets raises the concern of potential side effects. Given the intricate regulatory networks in which these miRNAs participate, targeting multiple genes may lead to unintended consequences, impacting cellular processes beyond the intended scope. Therefore, it is crucial to consider and discuss the possibility of side effects when exploring treatments based on miRNAs with diverse target profiles.

In conclusion, the present study shows, for the first time, that miR-3940-5p strongly suppresses the production of Aβ by specifically targeting *PSEN1*, the catalytic core subunit of γ-secretase. Our study is the first to link miR-3940-5p with AD and suggest miR-3940-5p could have potential to be a novel target in AD therapy.

## Data availability statement

The original contributions presented in the study are included in the article/Supplementary materials, further inquiries can be directed to the corresponding author.

## Ethics statement

Ethical approval was not required for the studies on humans in accordance with the local legislation and institutional requirements because only commercially available established cell lines were used.

## Author contributions

YQ: Data curation, Investigation, Methodology, Resources, Writing – original draft. XW: Funding acquisition, Supervision, Writing – review & editing. XG: Conceptualization, Funding acquisition, Investigation, Resources, Writing – review & editing.
