# Supplementary figures and images for: miR-3940-5p reduces amyloid β production via selectively targeting PSEN1
Source: Front Aging Neurosci. 2024 Mar 4;16:1346978. doi: 10.3389/fnagi.2024.1346978 (PMC10944889; doi:10.3389/fnagi.2024.1346978)

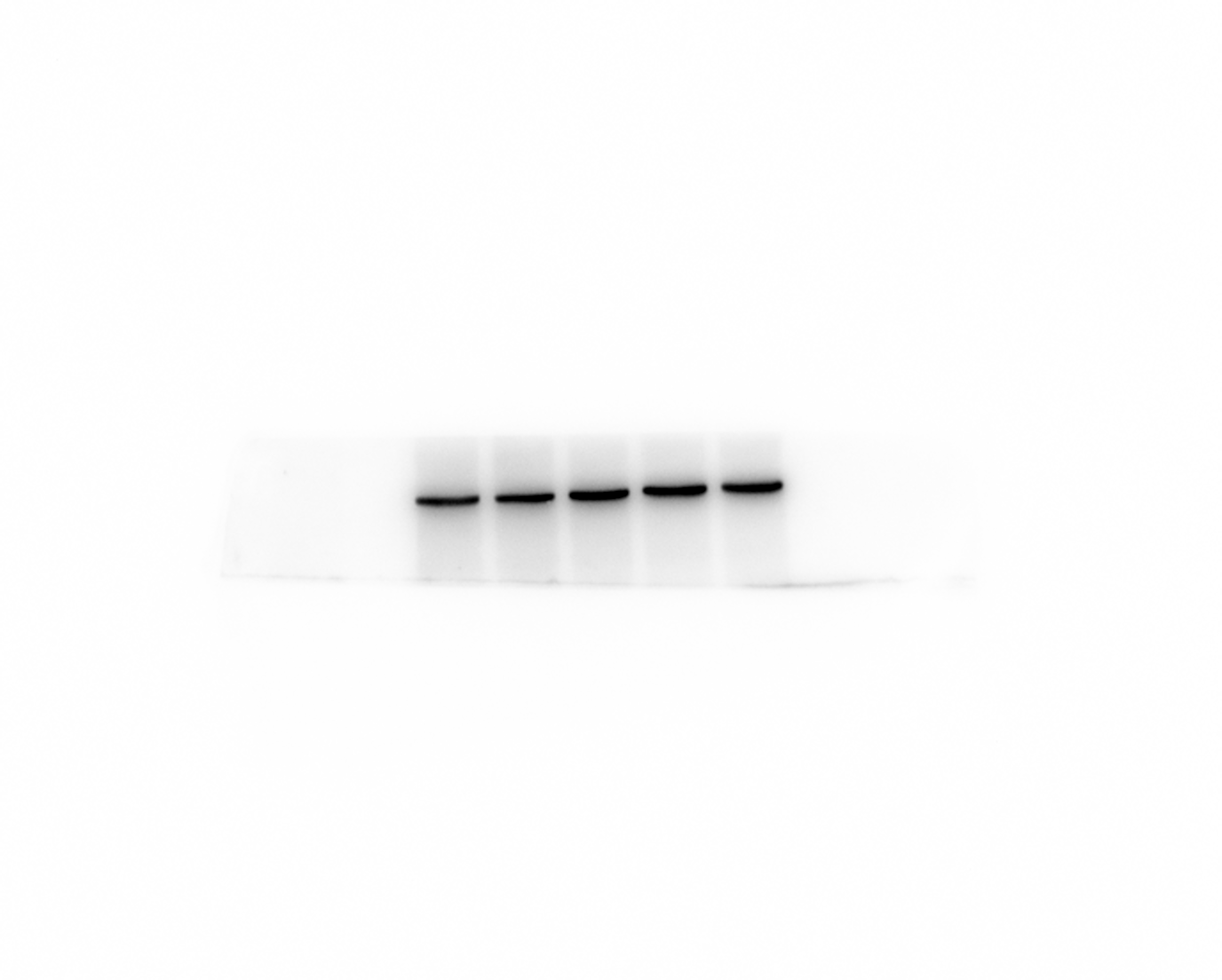

Supplement: Supplementary file 1 [file Data_Sheet_1.ZIP › raw data/fig2/fig2c-d/fig2c-GAPDH.tif]

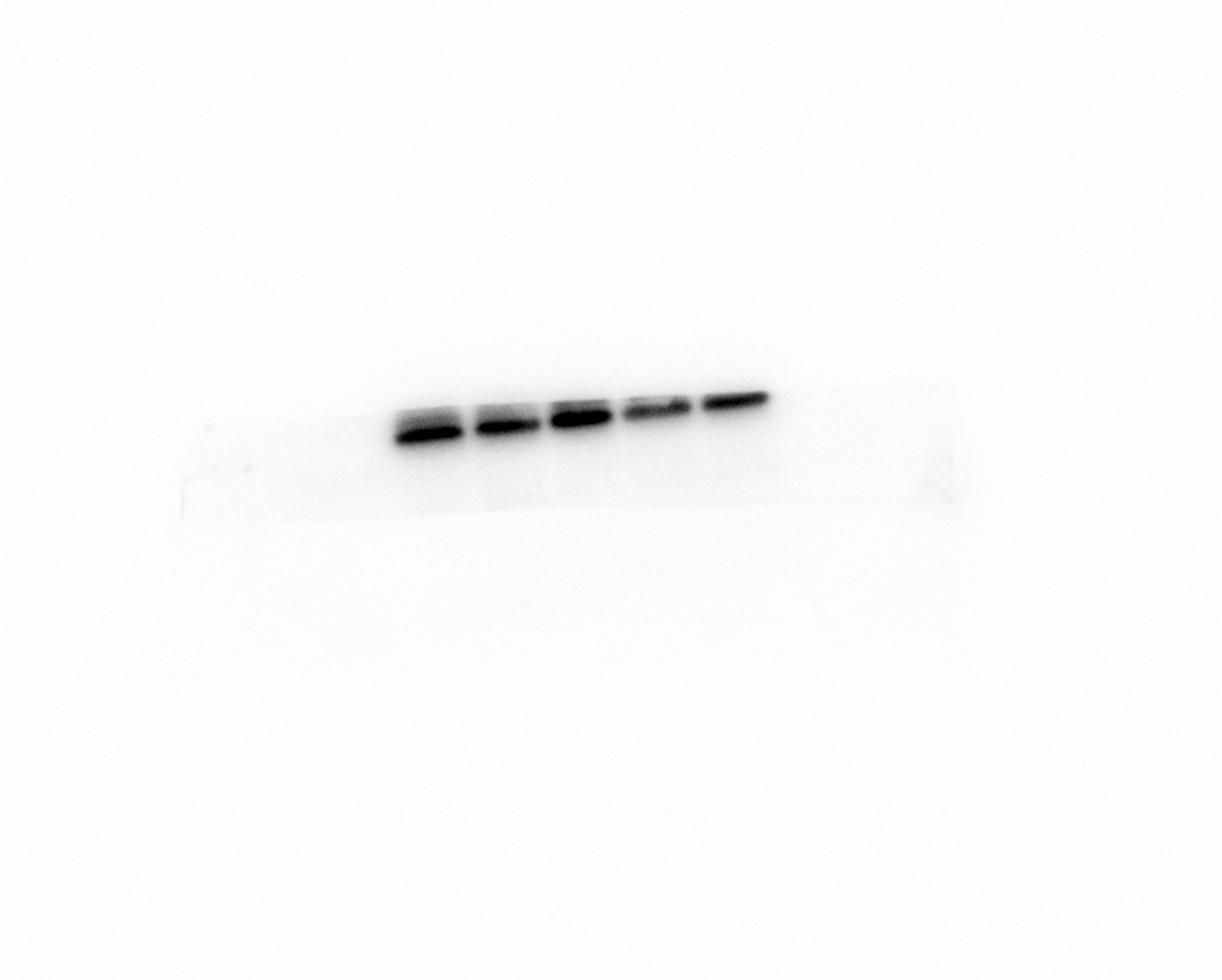

Supplement: Supplementary file 1 [file Data_Sheet_1.ZIP › raw data/fig2/fig2c-d/fig2c-PSEN1.tif]

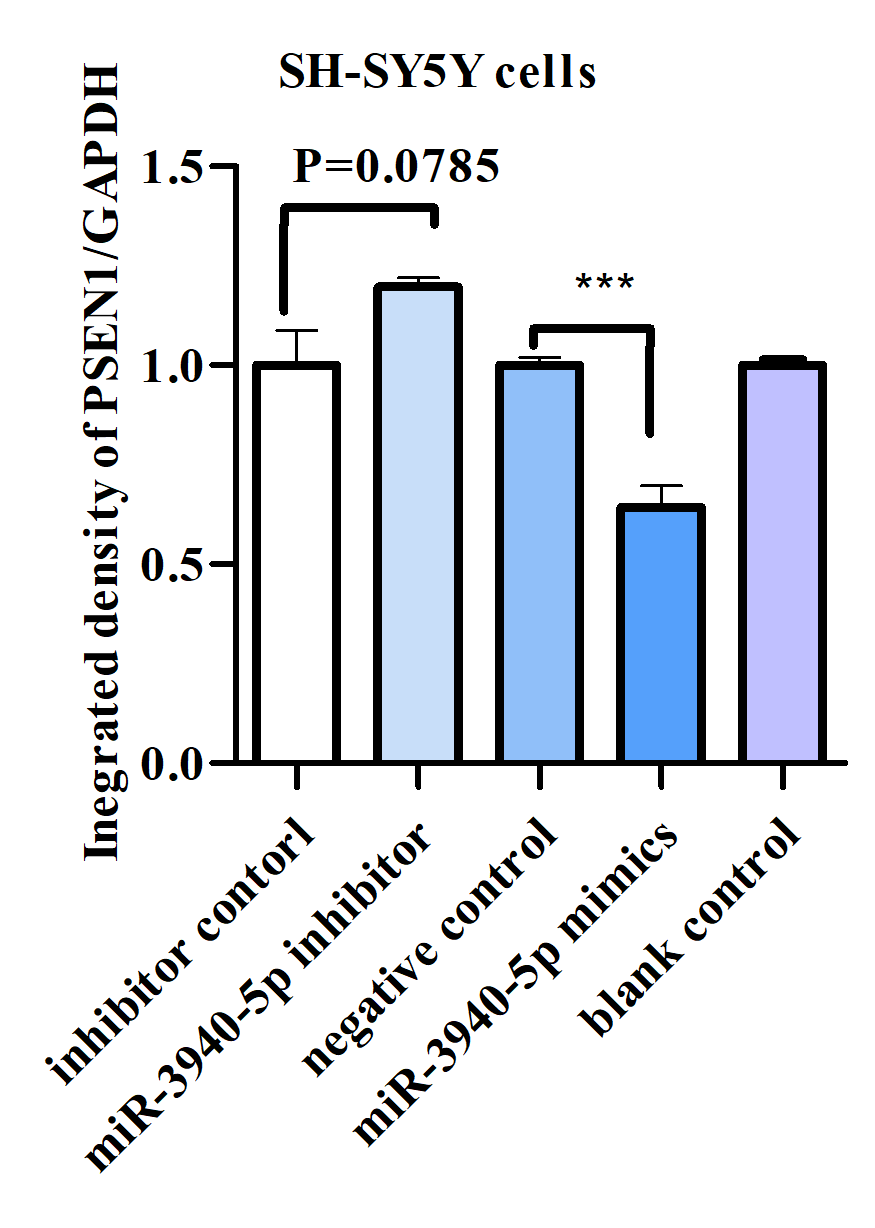

Supplement: Supplementary file 1 [file Data_Sheet_1.ZIP › raw data/fig2/fig2c-d/fig2d.tif]

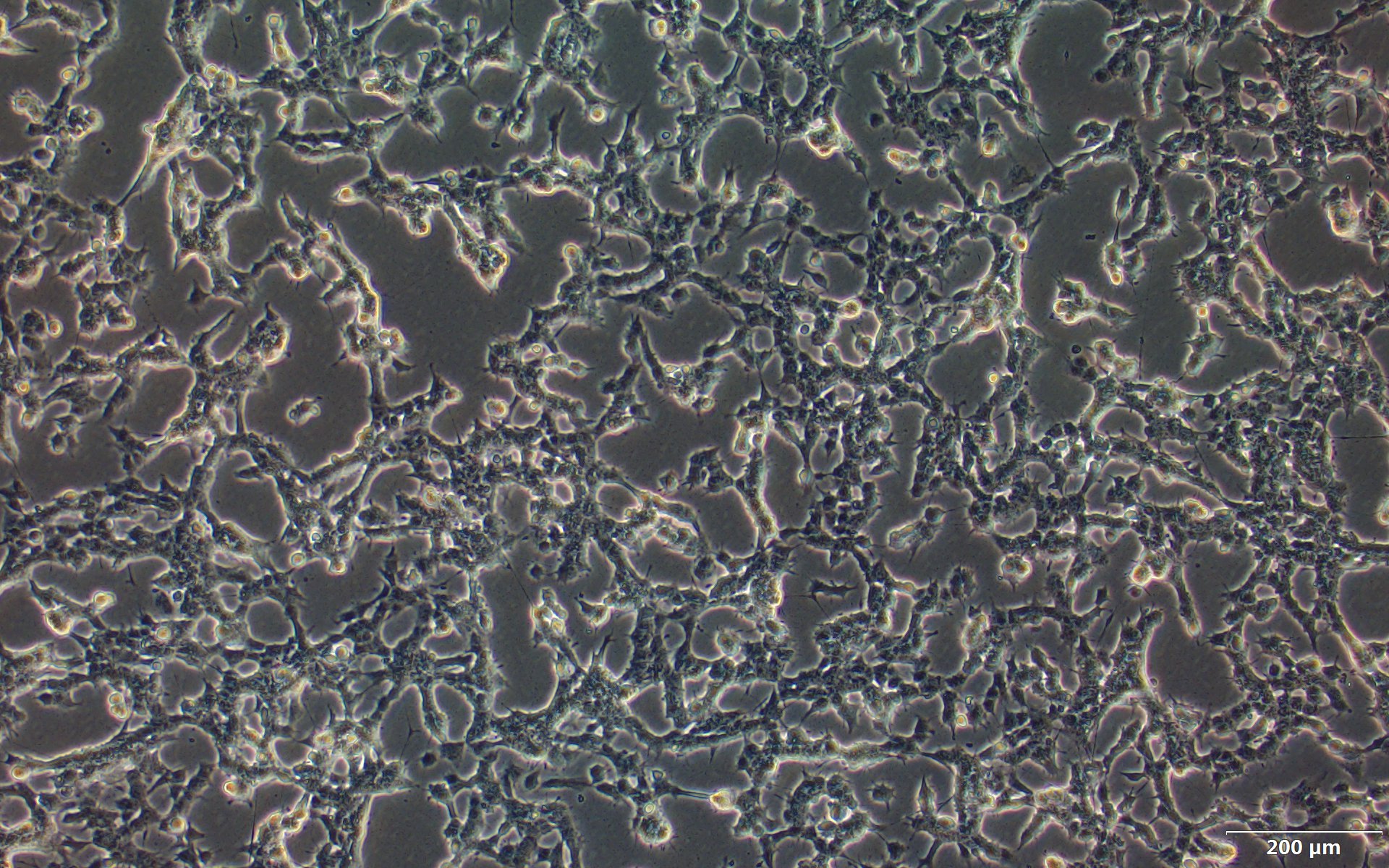

Supplement: Supplementary file 1 [file Data_Sheet_1.ZIP › raw data/fig3/fig3a/SH-SY5Y-BF.jpg]

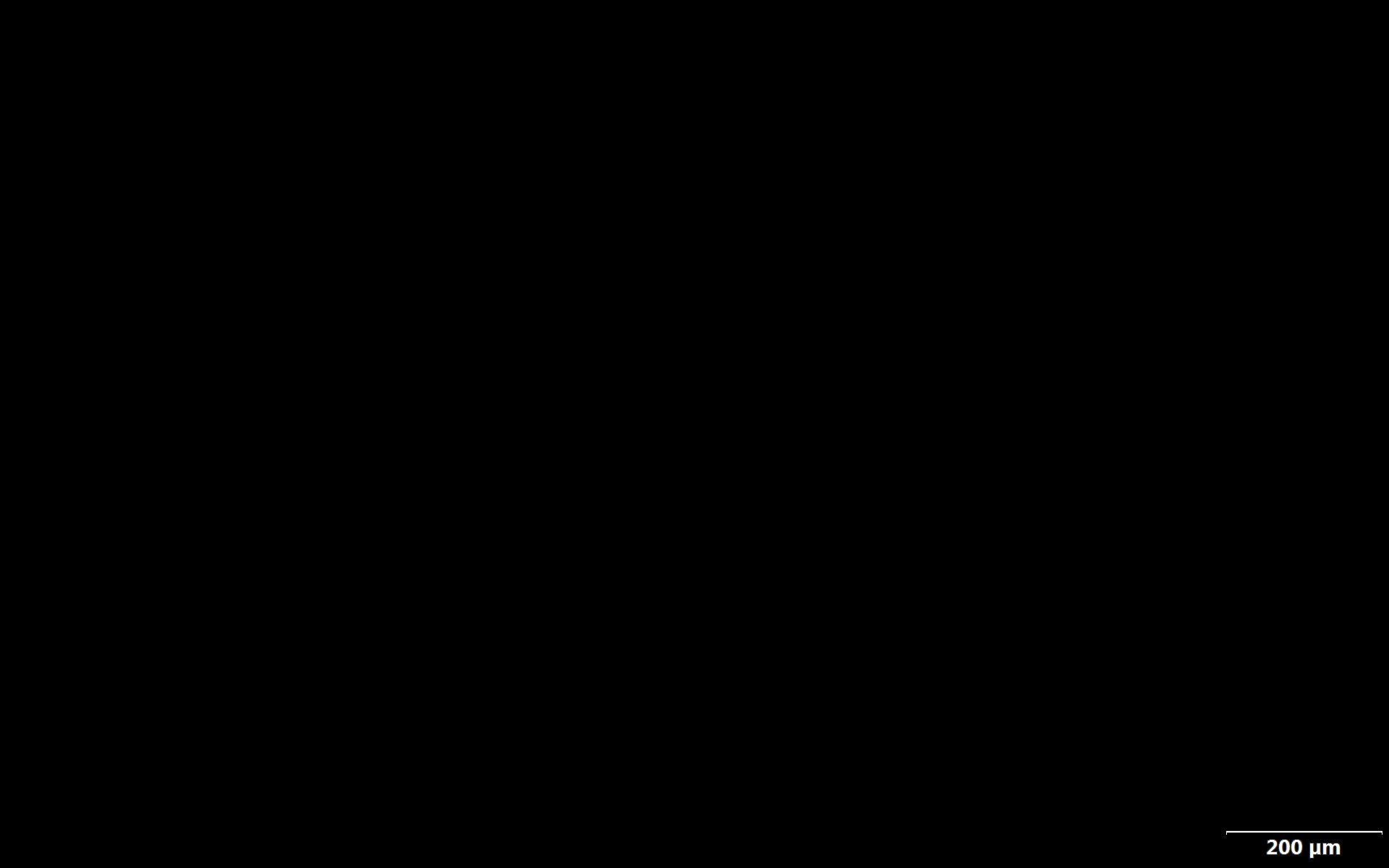

Supplement: Supplementary file 1 [file Data_Sheet_1.ZIP › raw data/fig3/fig3a/SH-SY5Y-Flu.jpg]

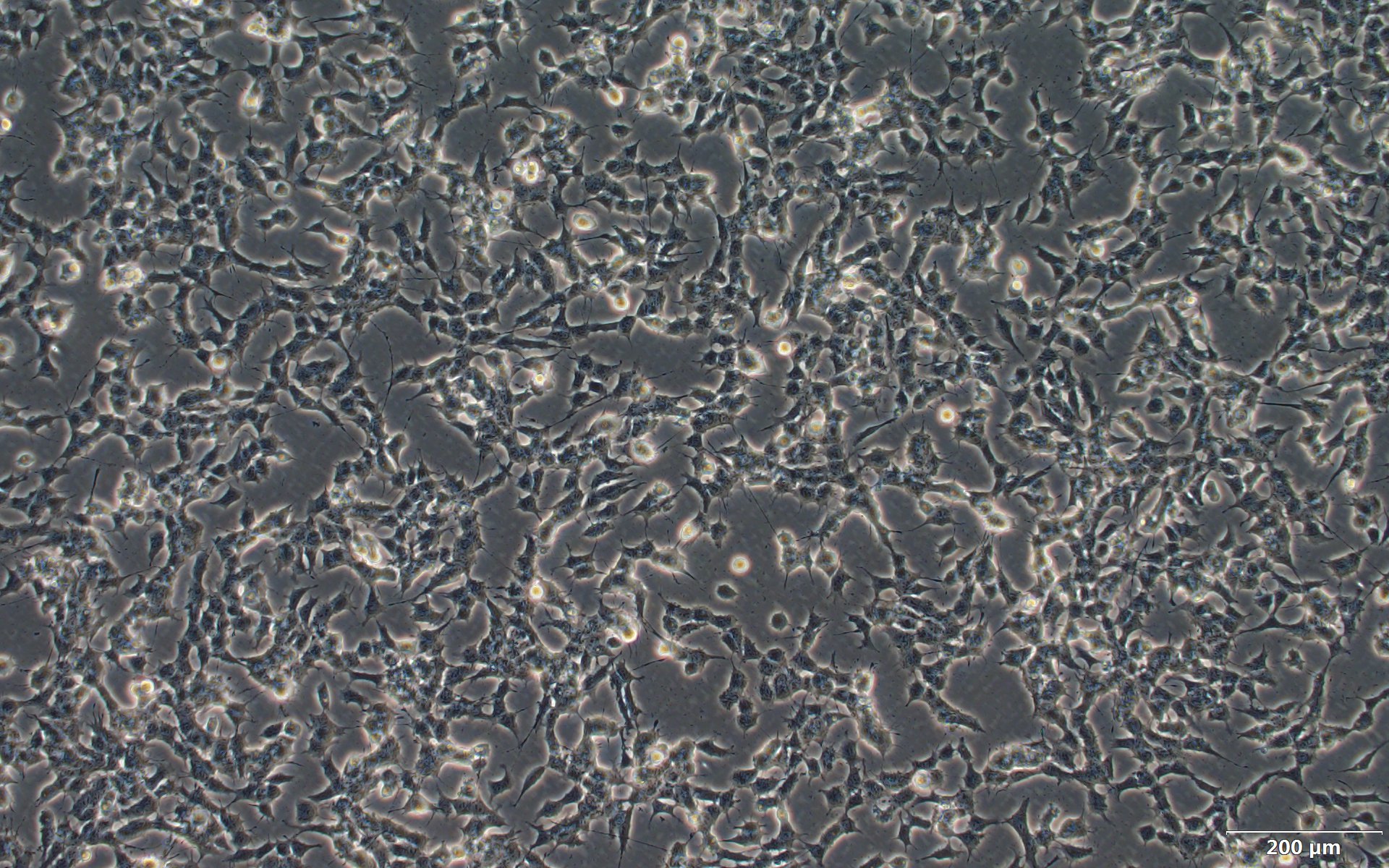

Supplement: Supplementary file 1 [file Data_Sheet_1.ZIP › raw data/fig3/fig3a/SH-SY5Y-LV-miR-3940-5p-pre-BF.jpg]

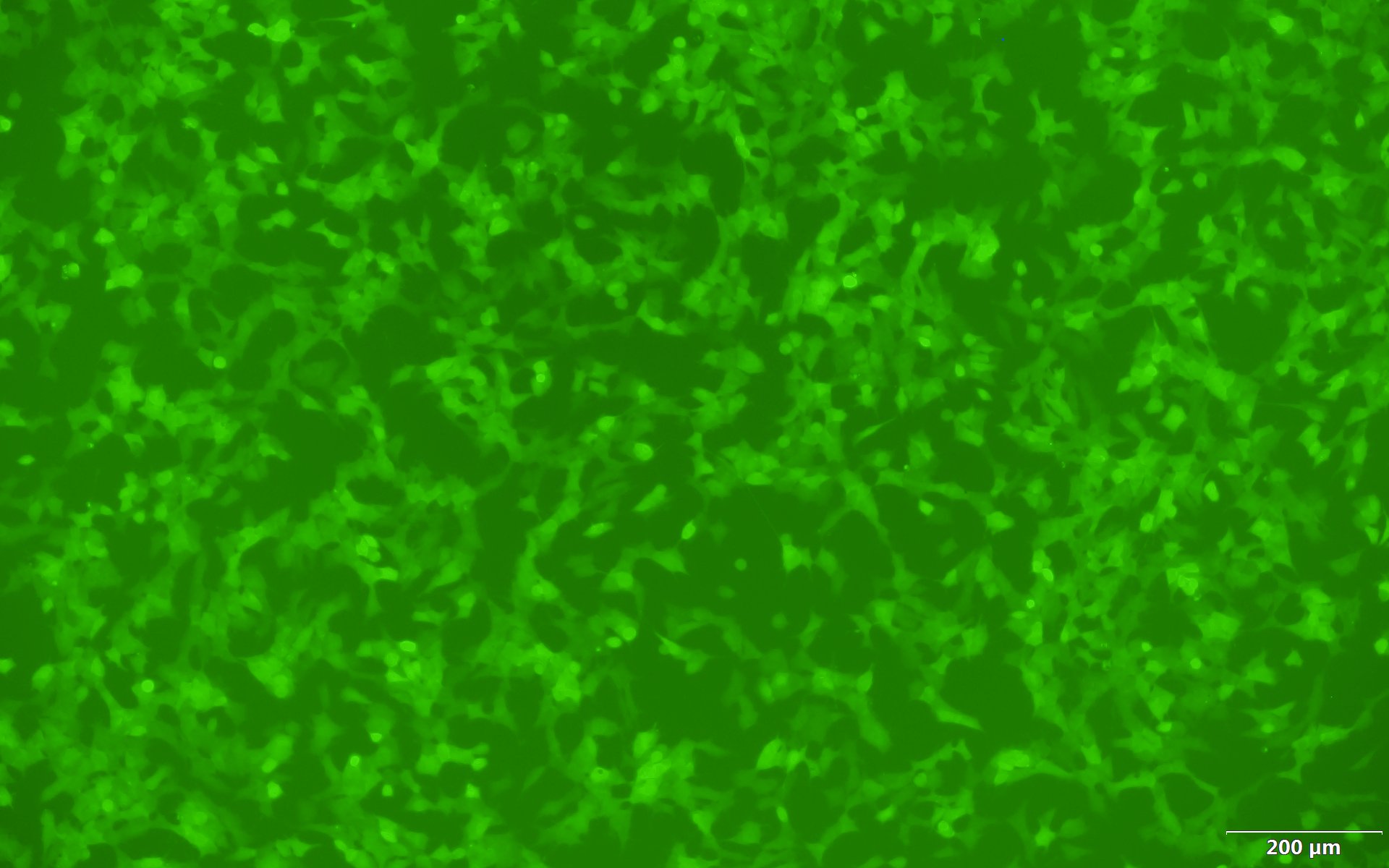

Supplement: Supplementary file 1 [file Data_Sheet_1.ZIP › raw data/fig3/fig3a/SH-SY5Y-LV-miR-3940-5p-pre-Flu.jpg]

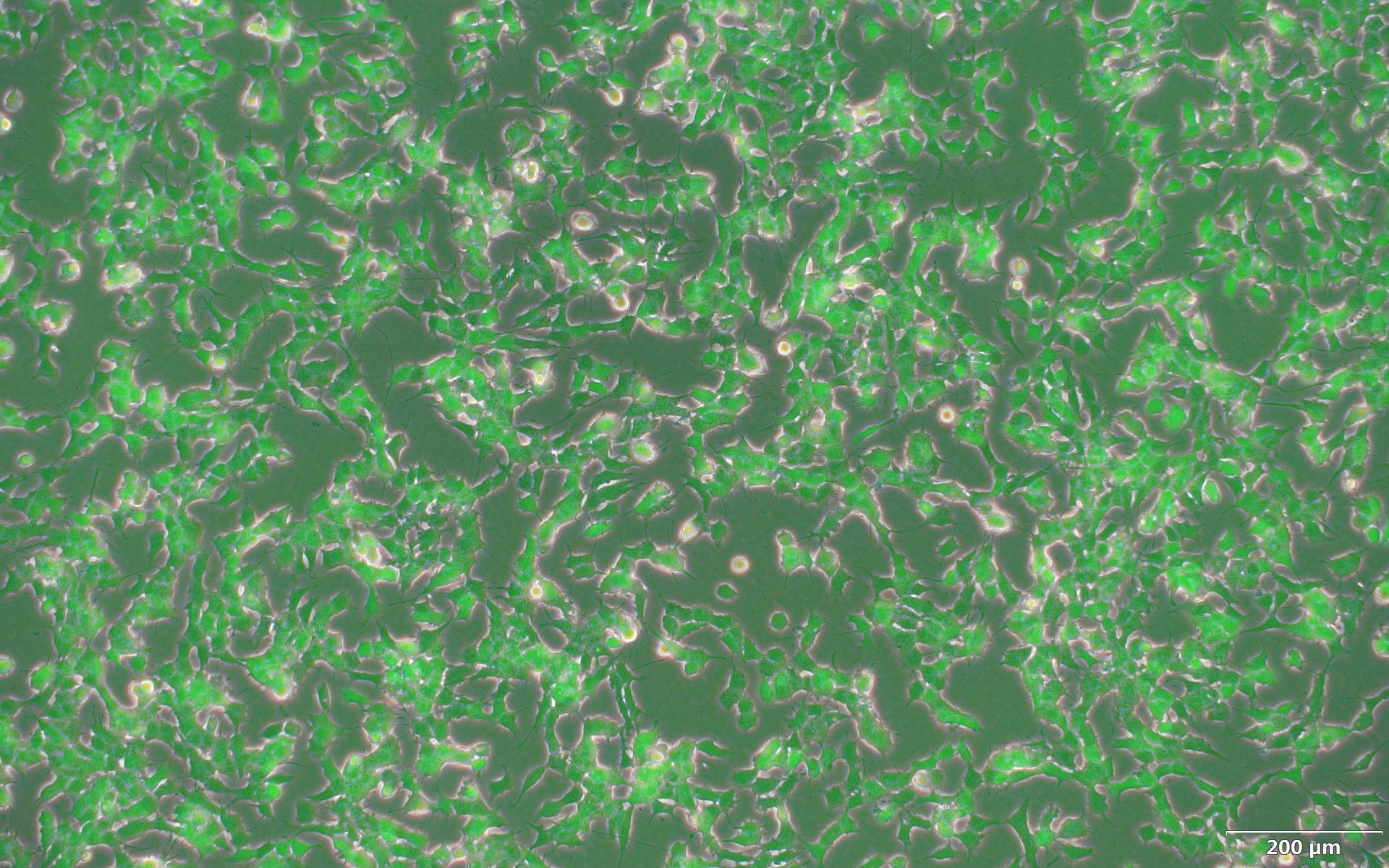

Supplement: Supplementary file 1 [file Data_Sheet_1.ZIP › raw data/fig3/fig3a/SH-SY5Y-LV-miR-3940-5p-pre-merge.jpg]

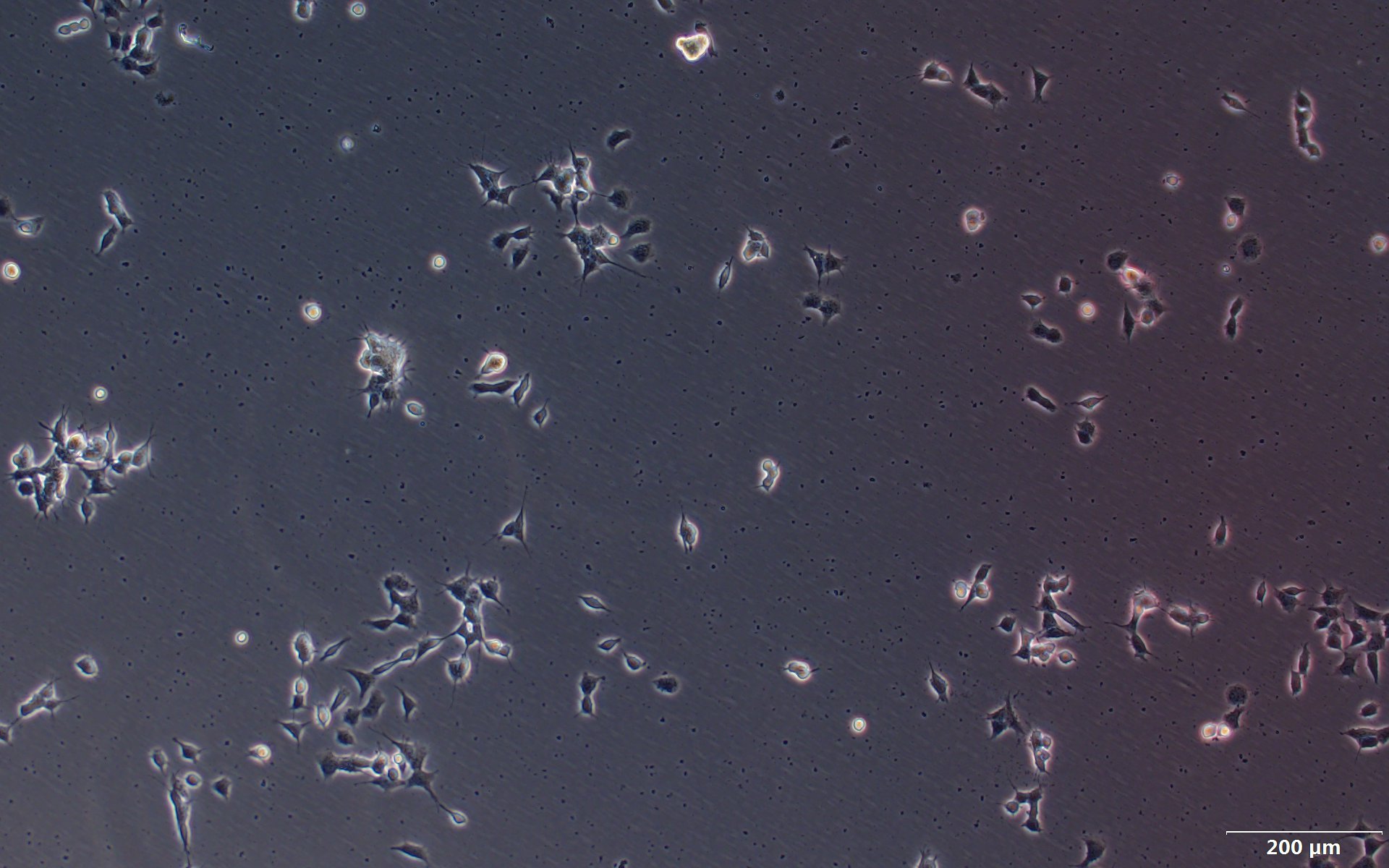

Supplement: Supplementary file 1 [file Data_Sheet_1.ZIP › raw data/fig3/fig3a/SH-SY5Y-LV-miR-NC-BF.jpg]

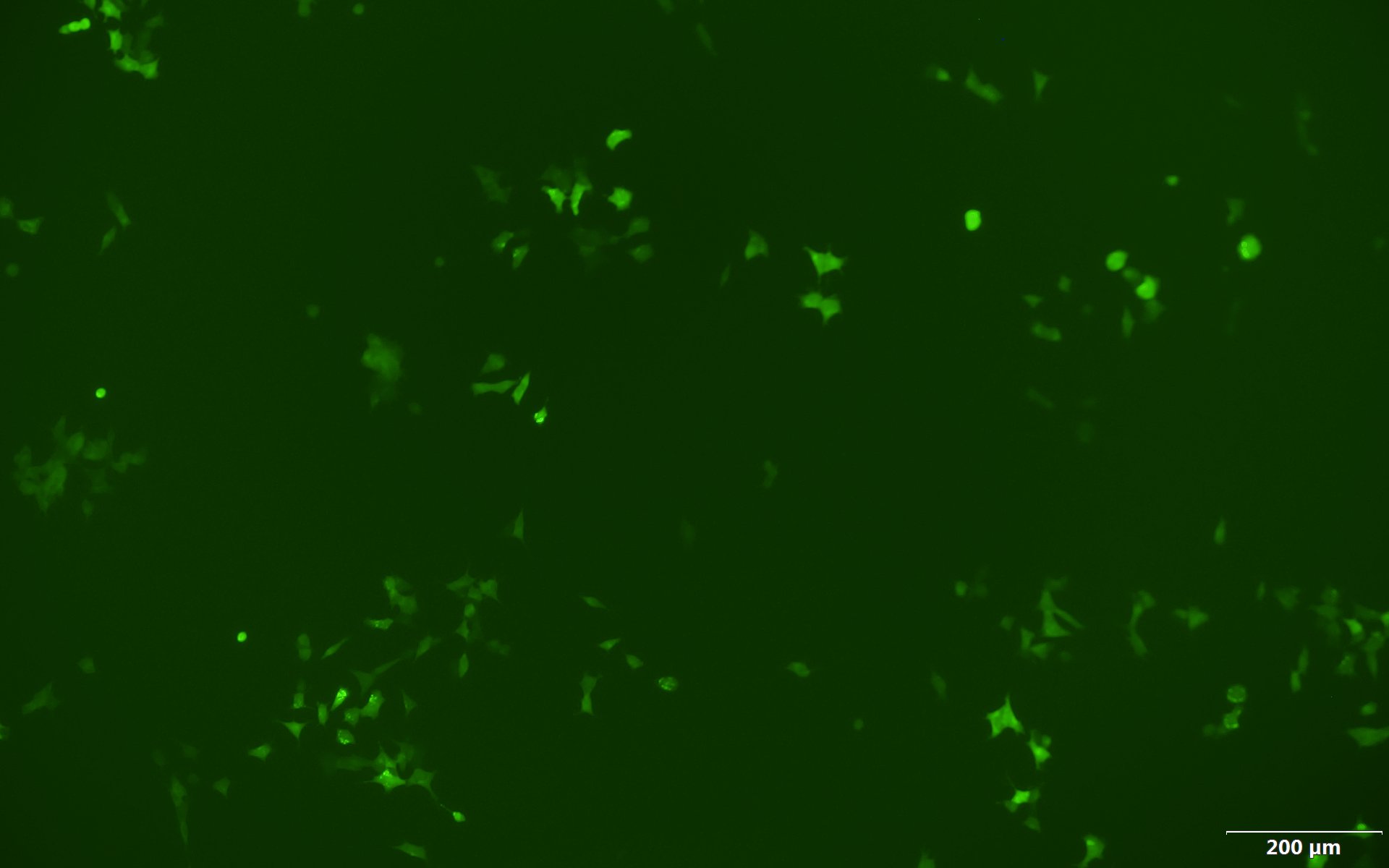

Supplement: Supplementary file 1 [file Data_Sheet_1.ZIP › raw data/fig3/fig3a/SH-SY5Y-LV-miR-NC-Flu.jpg]

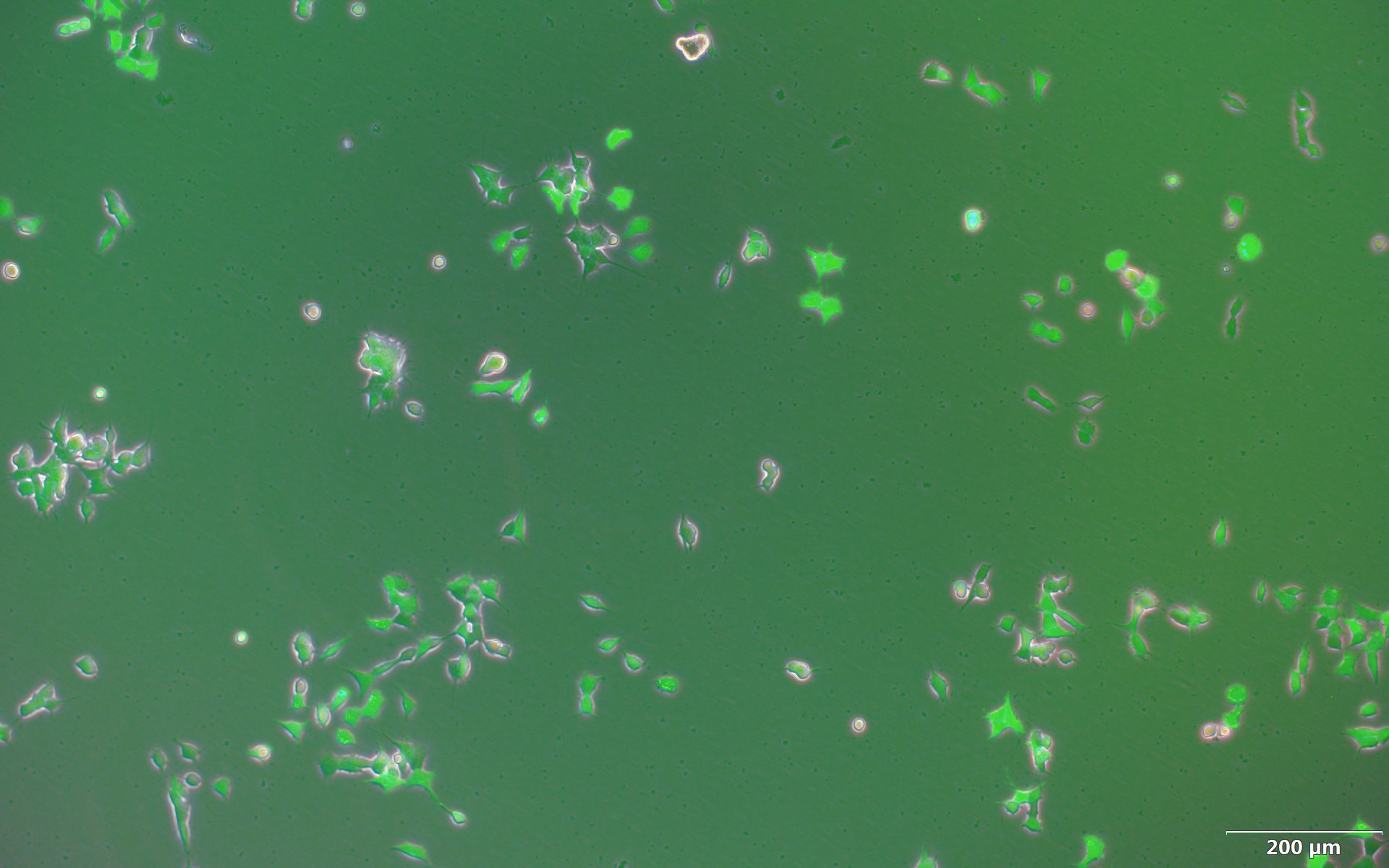

Supplement: Supplementary file 1 [file Data_Sheet_1.ZIP › raw data/fig3/fig3a/SH-SY5Y-LV-miR-NC-merge.jpg]

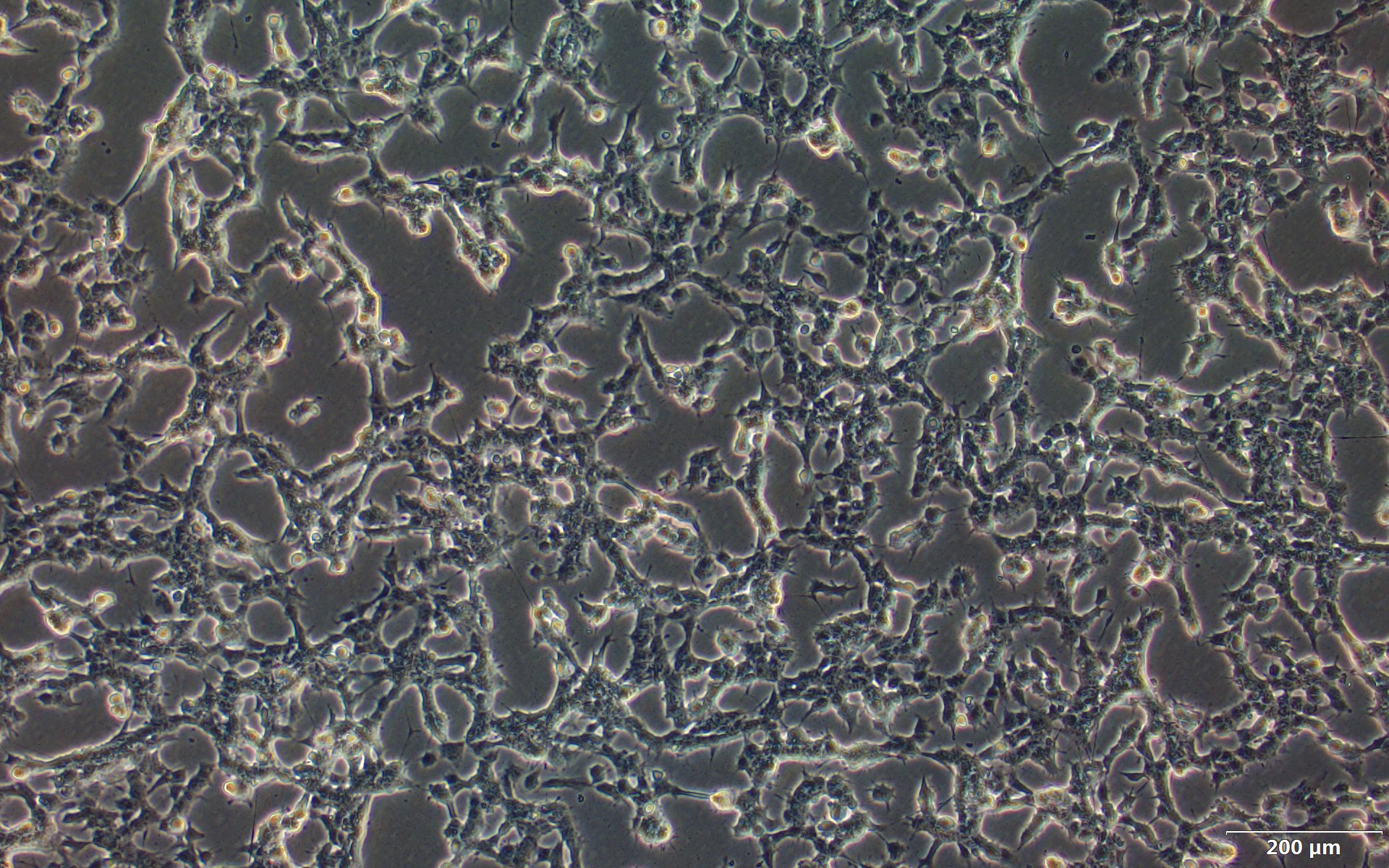

Supplement: Supplementary file 1 [file Data_Sheet_1.ZIP › raw data/fig3/fig3a/SH-SY5Y-merge.jpg]

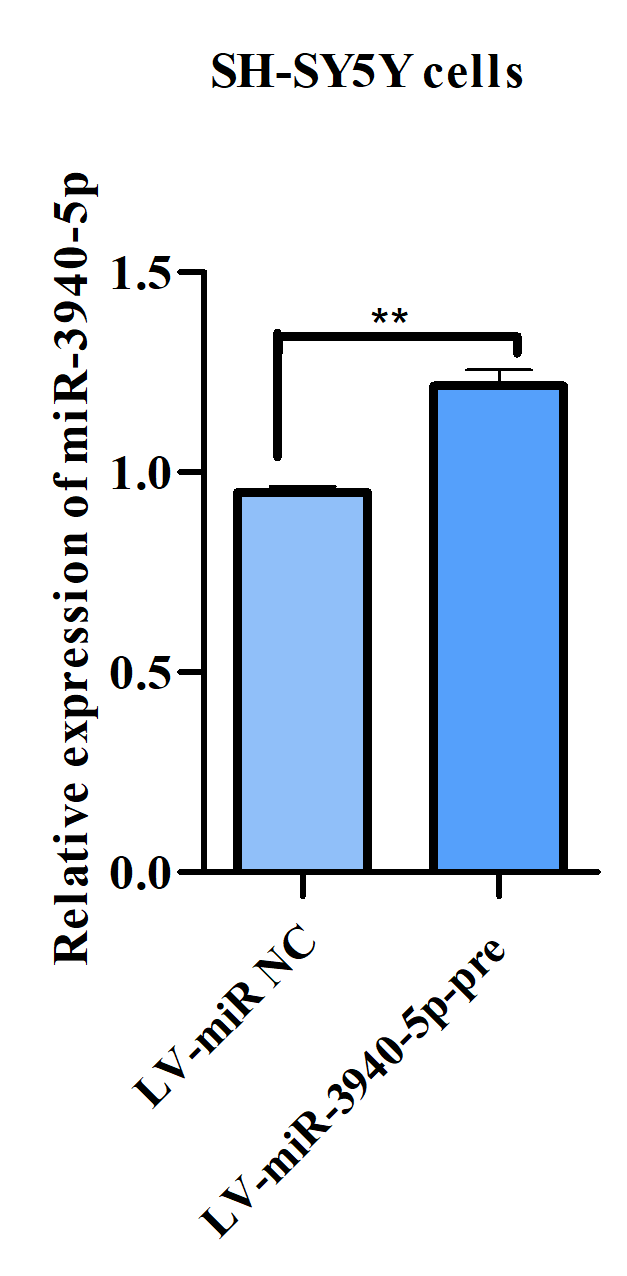

Supplement: Supplementary file 1 [file Data_Sheet_1.ZIP › raw data/fig3/fig3b/fig3b.tif]

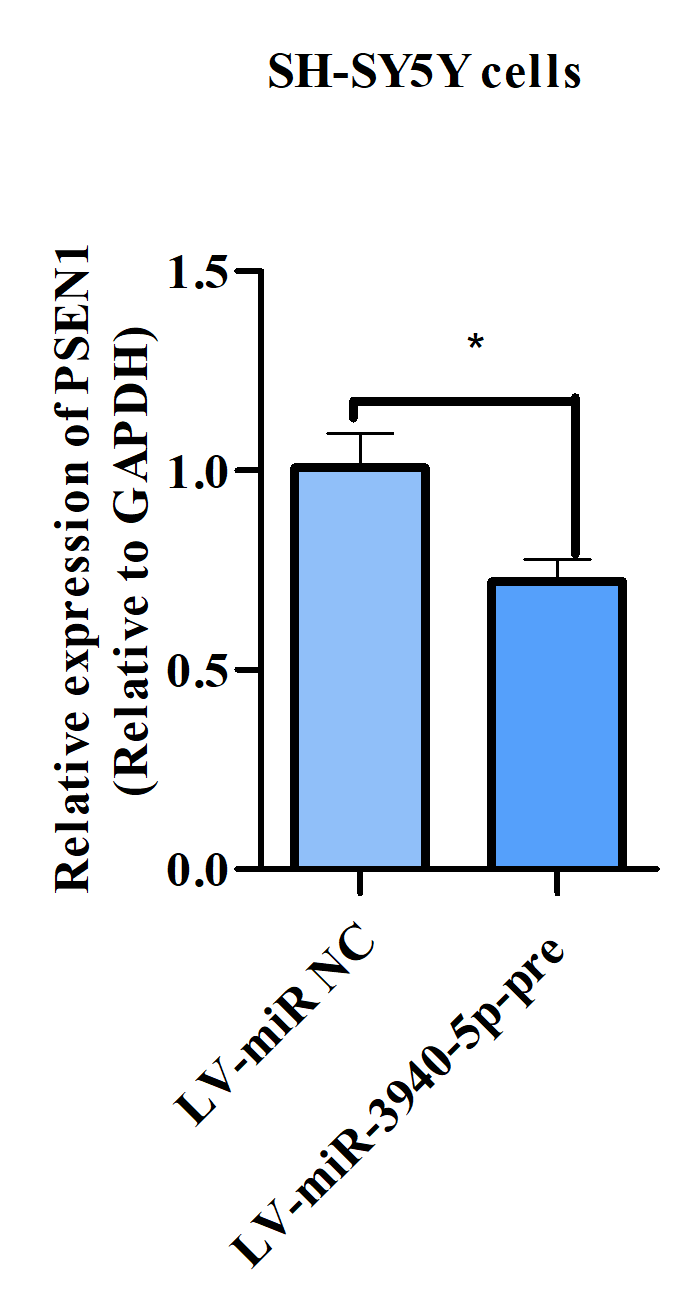

Supplement: Supplementary file 1 [file Data_Sheet_1.ZIP › raw data/fig3/fig3c/fig3c.tif]

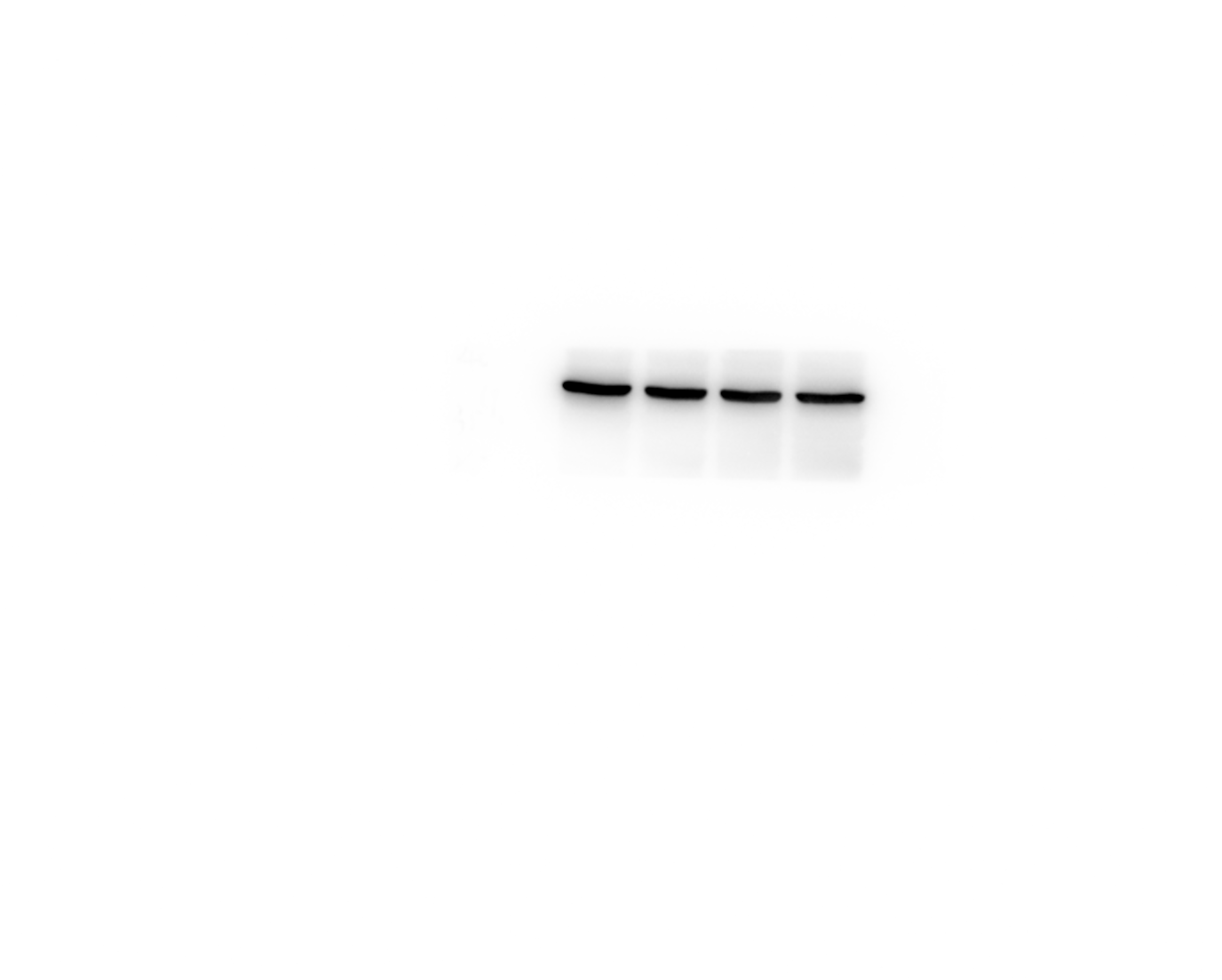

Supplement: Supplementary file 1 [file Data_Sheet_1.ZIP › raw data/fig3/fig3d-e/fig3d-GAPDH.tif]

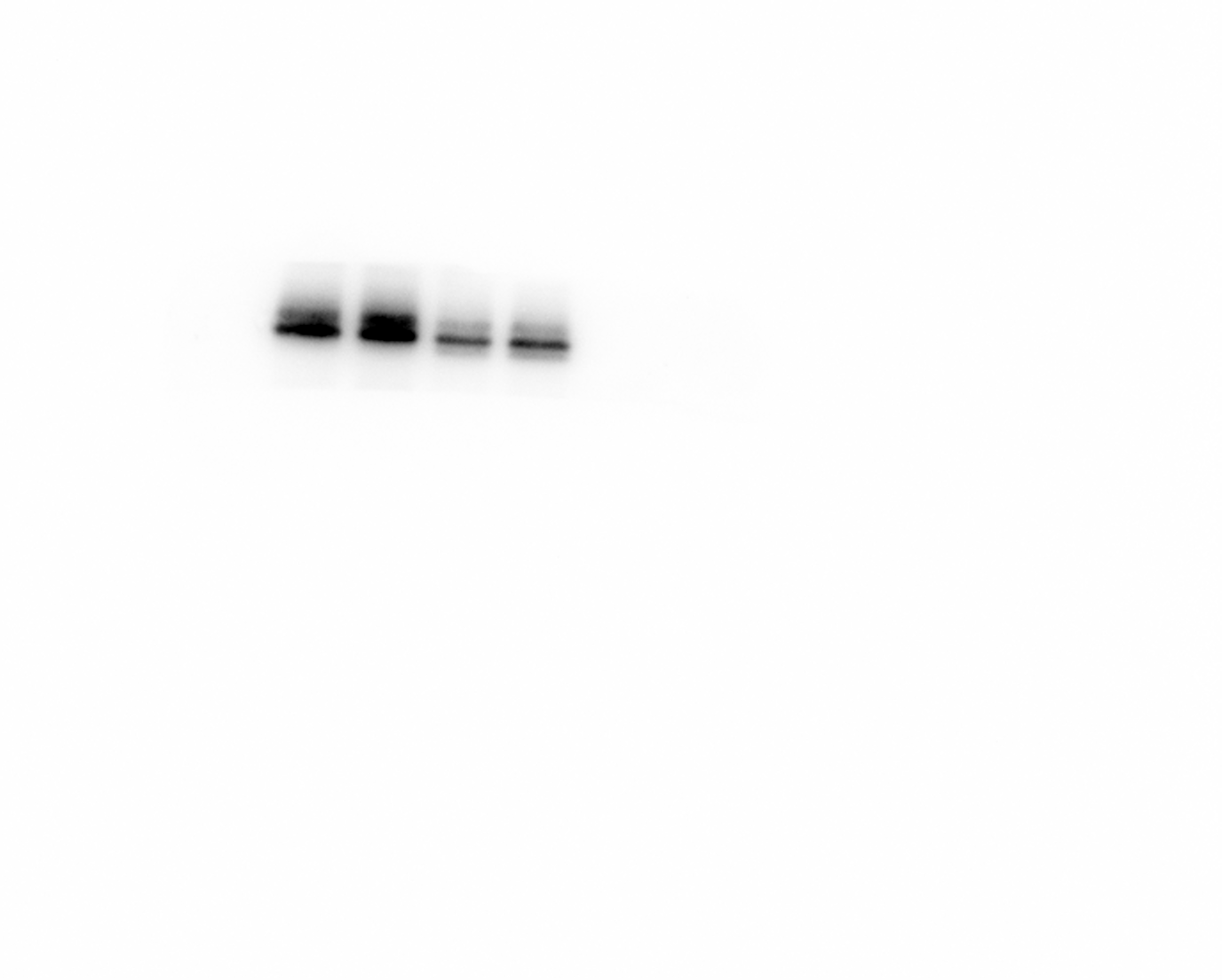

Supplement: Supplementary file 1 [file Data_Sheet_1.ZIP › raw data/fig3/fig3d-e/fig3d-PSEN1.tif]

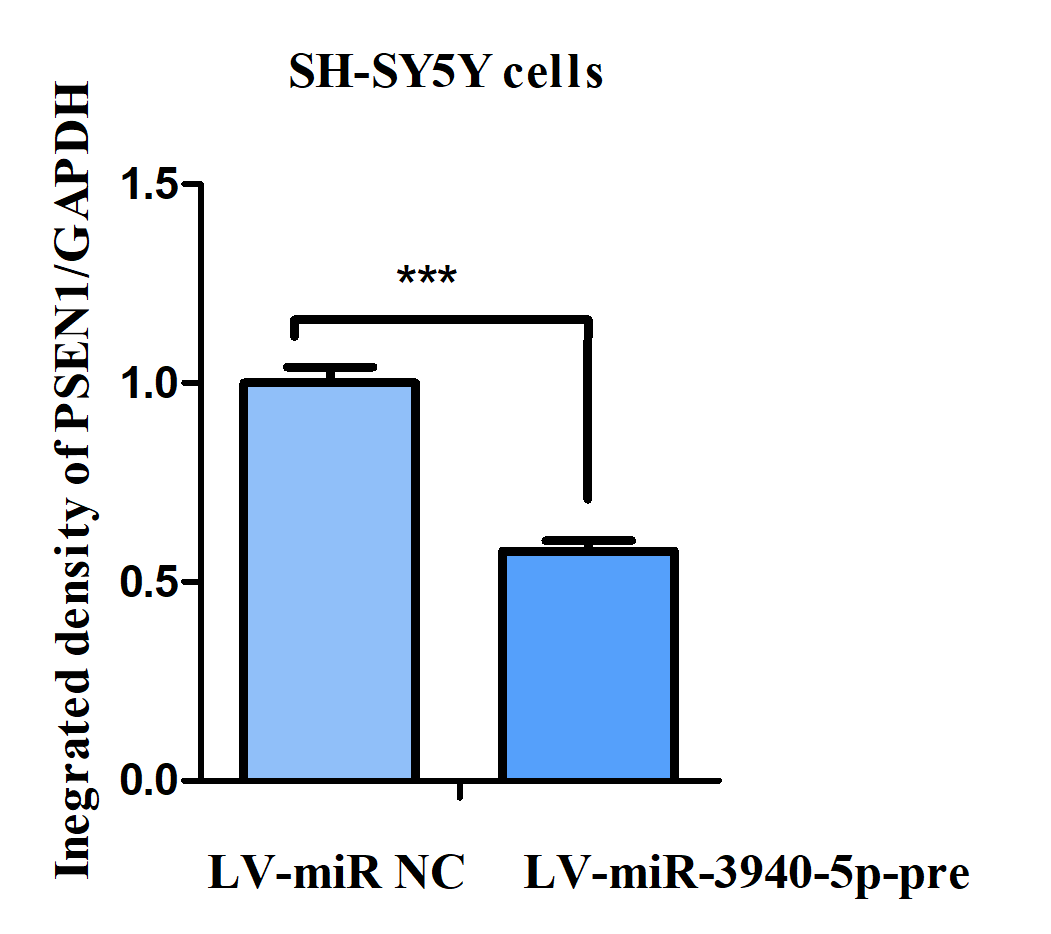

Supplement: Supplementary file 1 [file Data_Sheet_1.ZIP › raw data/fig3/fig3d-e/fig3e.tif]

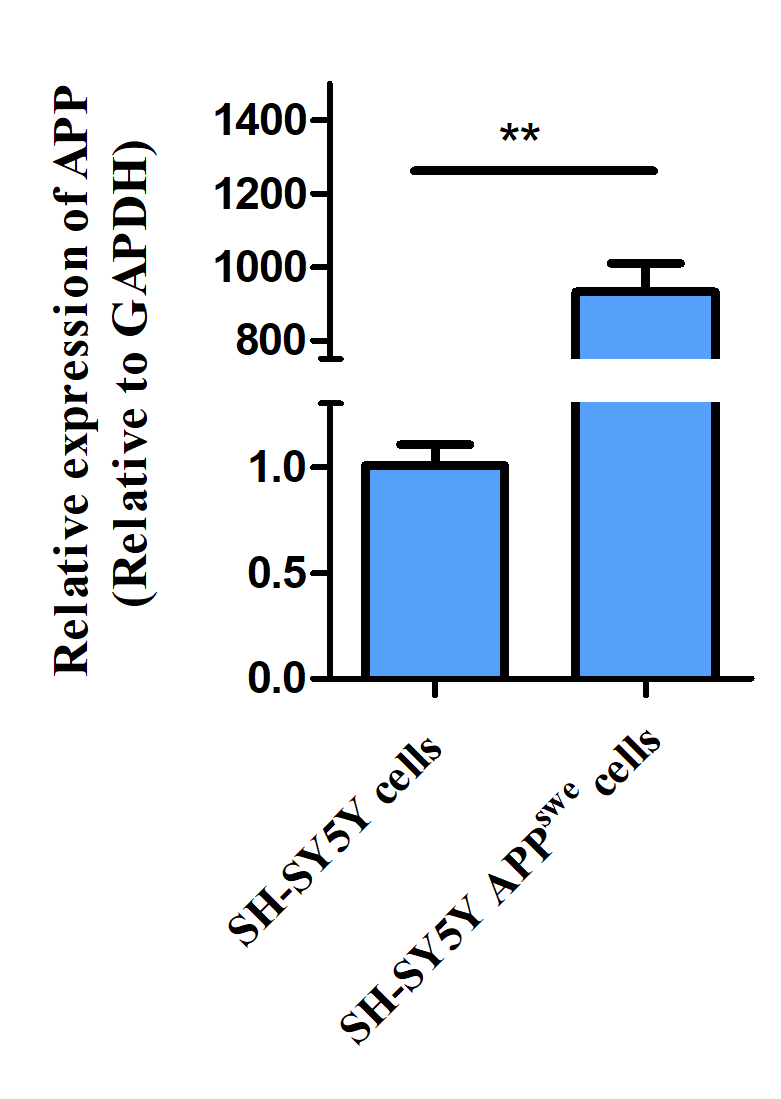

Supplement: Supplementary file 1 [file Data_Sheet_1.ZIP › raw data/fig4/fig4a/fig4a.tif]

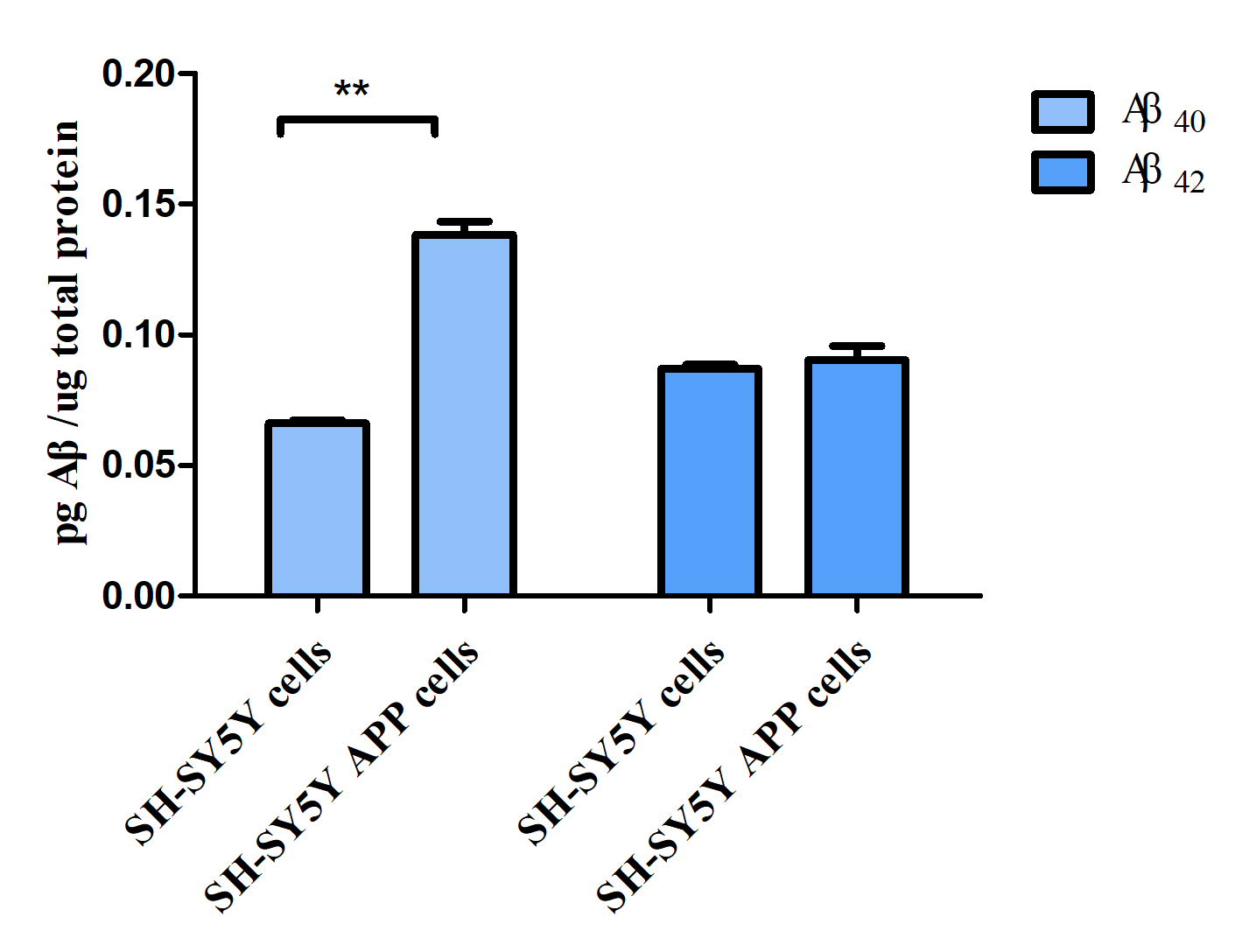

Supplement: Supplementary file 1 [file Data_Sheet_1.ZIP › raw data/fig4/fig4b/fig4b.tif]

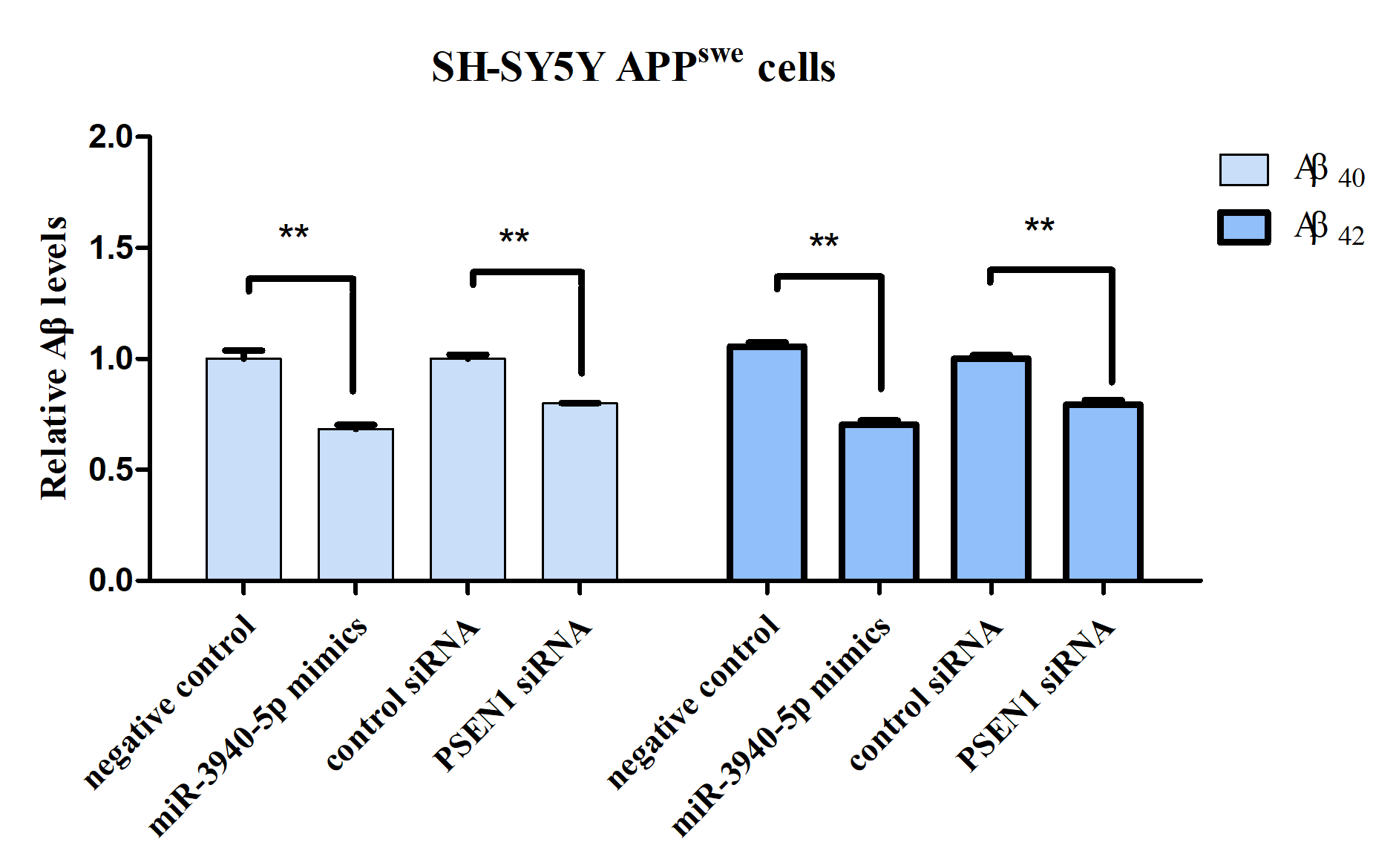

Supplement: Supplementary file 1 [file Data_Sheet_1.ZIP › raw data/fig4/fig4c/fig4c.tif]

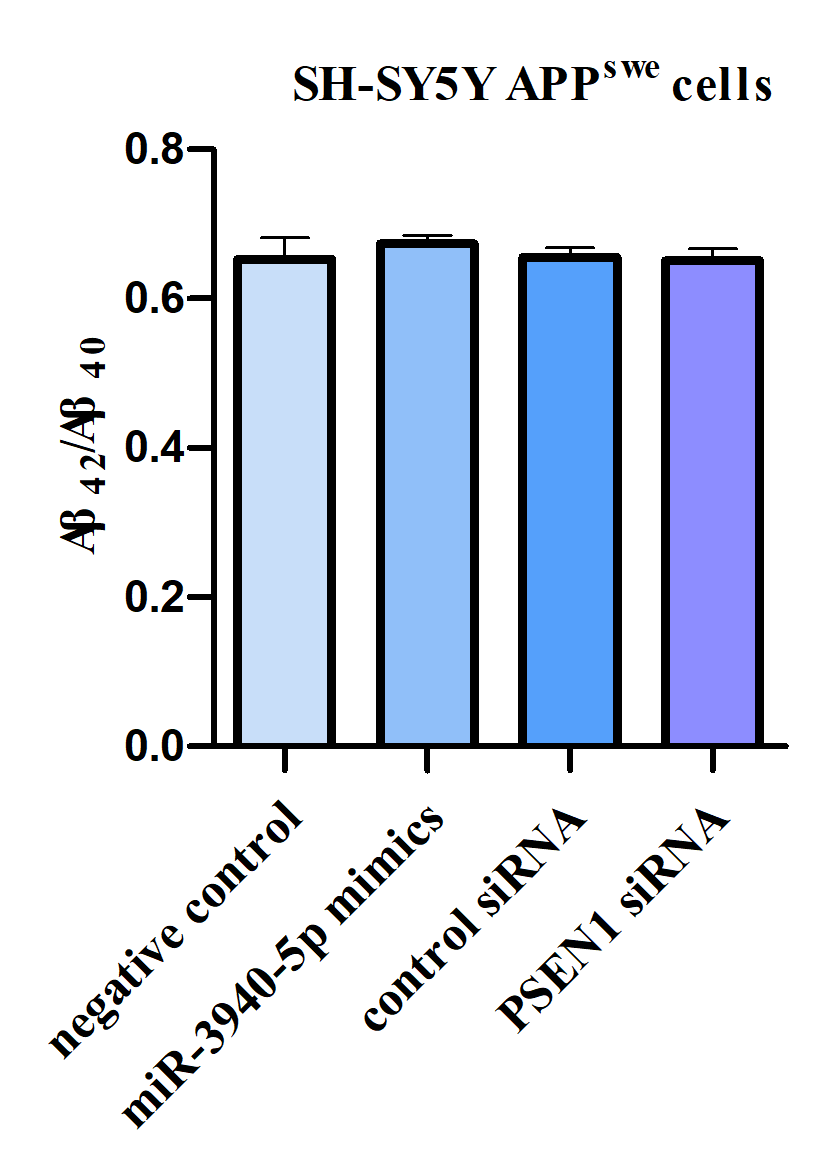

Supplement: Supplementary file 1 [file Data_Sheet_1.ZIP › raw data/fig4/fig4c/fig4d.tif]

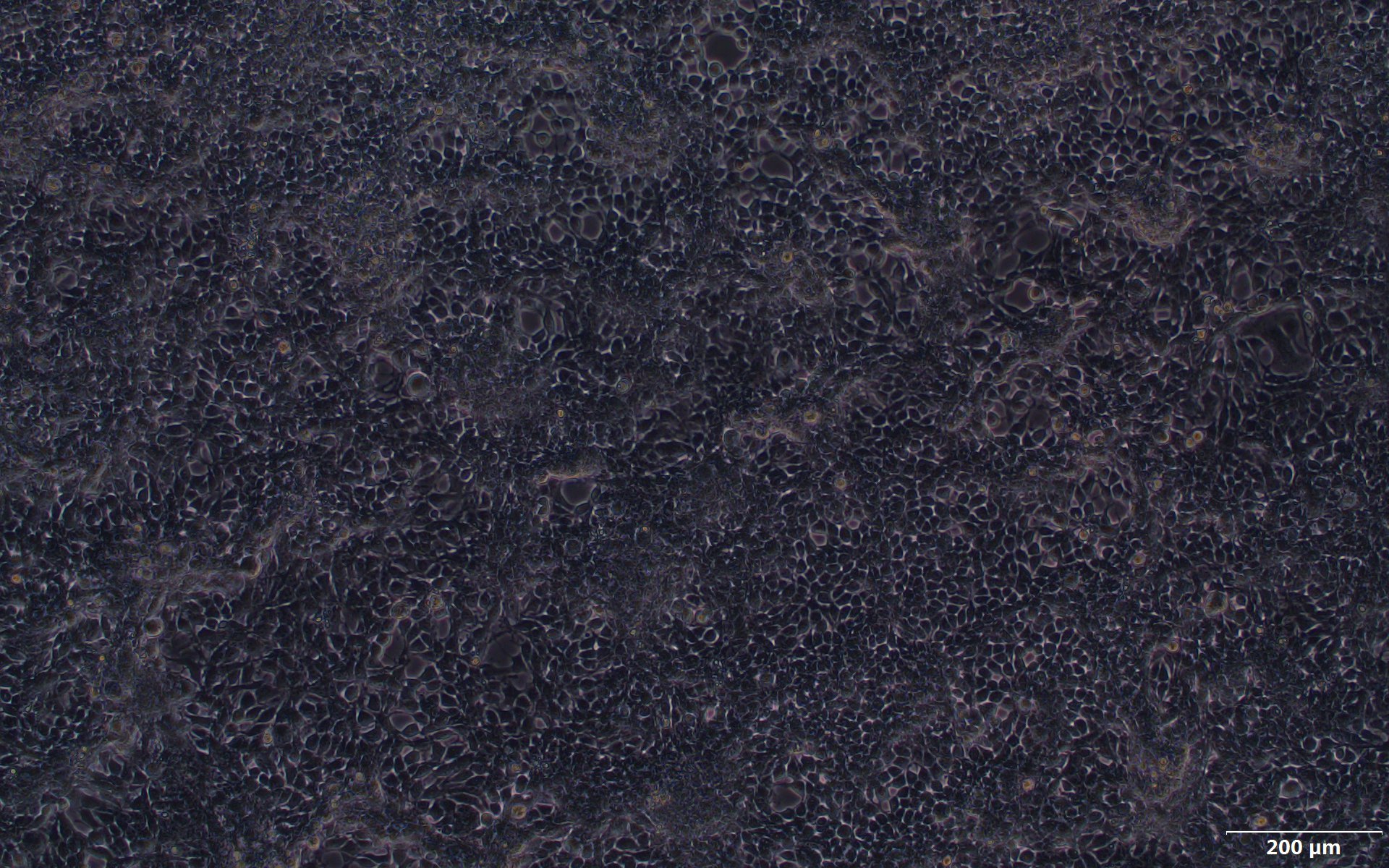

Supplement: Supplementary file 1 [file Data_Sheet_1.ZIP › raw data/fig4/fig4e/SH-SY5Y-APP-BF.jpg]

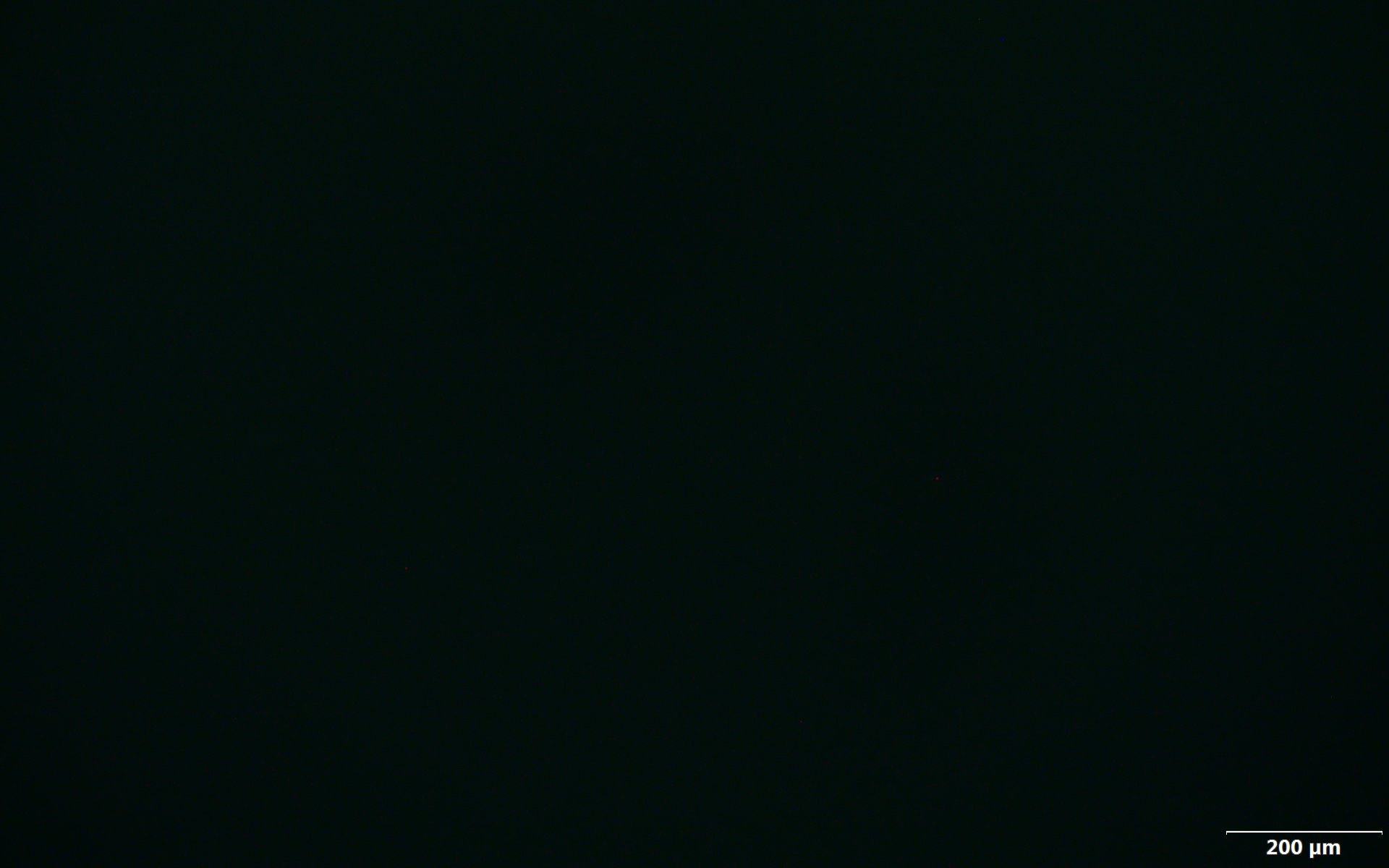

Supplement: Supplementary file 1 [file Data_Sheet_1.ZIP › raw data/fig4/fig4e/SH-SY5Y-APP-Flu.jpg]

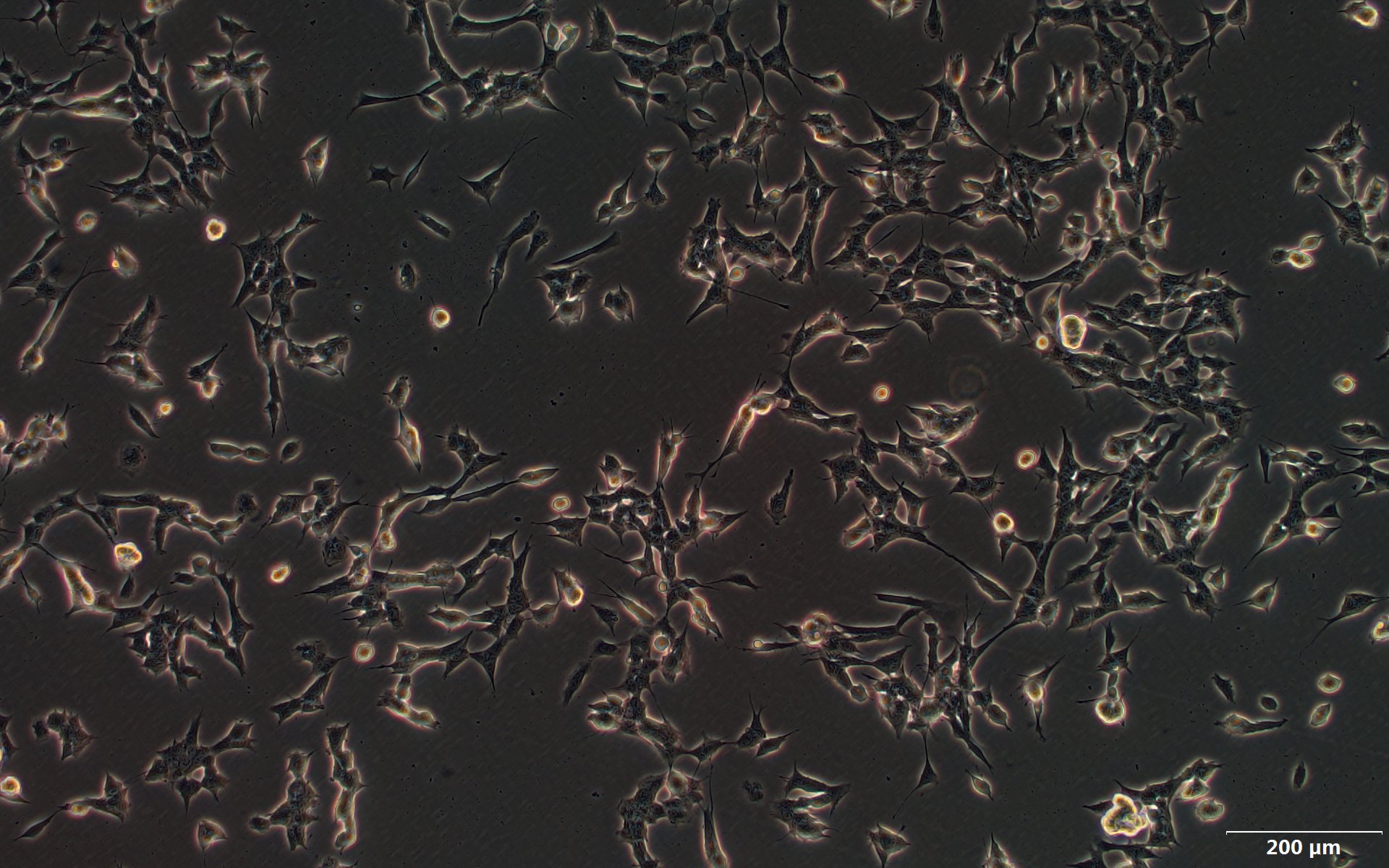

Supplement: Supplementary file 1 [file Data_Sheet_1.ZIP › raw data/fig4/fig4e/SH-SY5Y-APP-LV-miR-3940-5p-pre-BF.jpg]

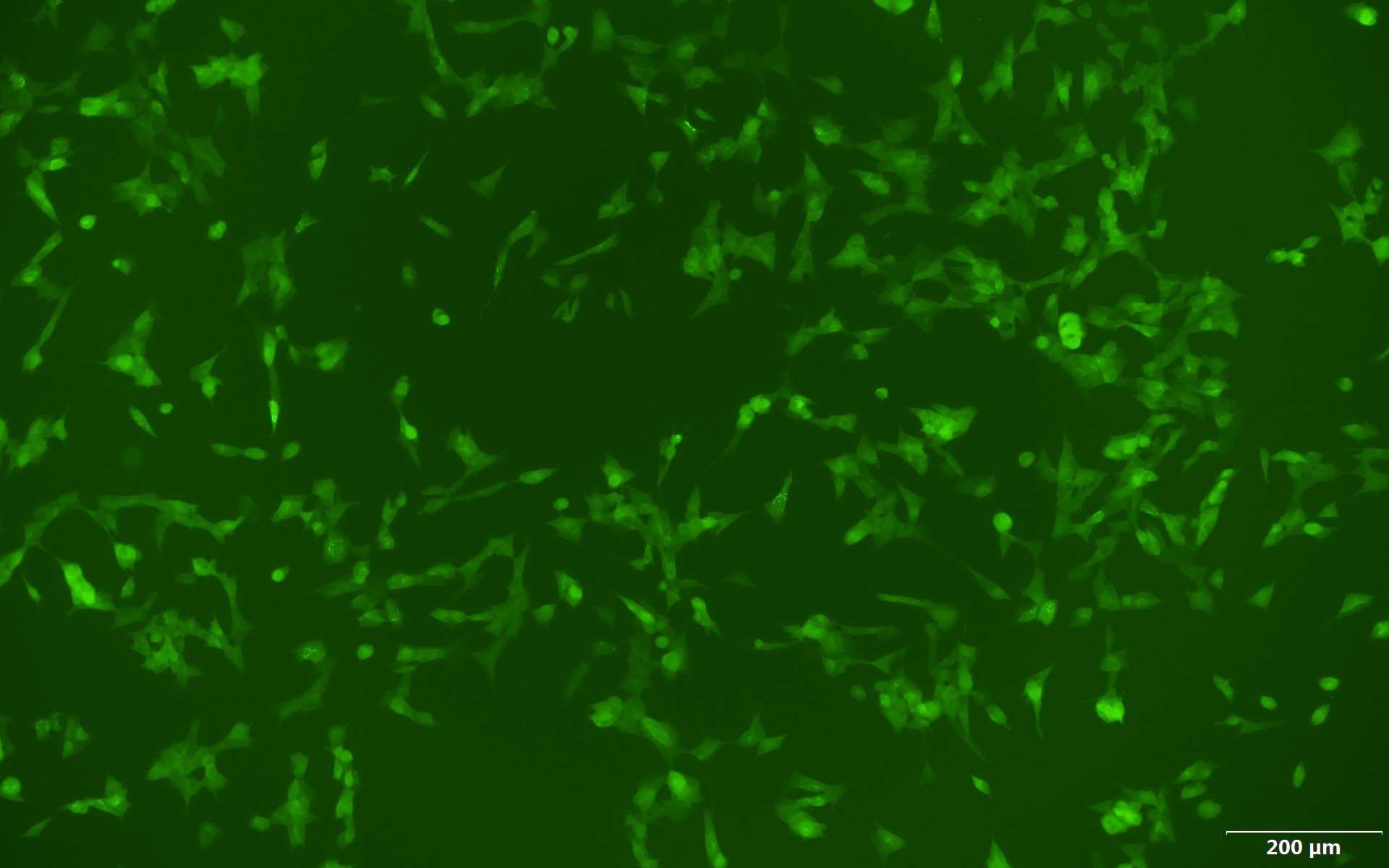

Supplement: Supplementary file 1 [file Data_Sheet_1.ZIP › raw data/fig4/fig4e/SH-SY5Y-APP-LV-miR-3940-5p-pre-Flu.jpg]

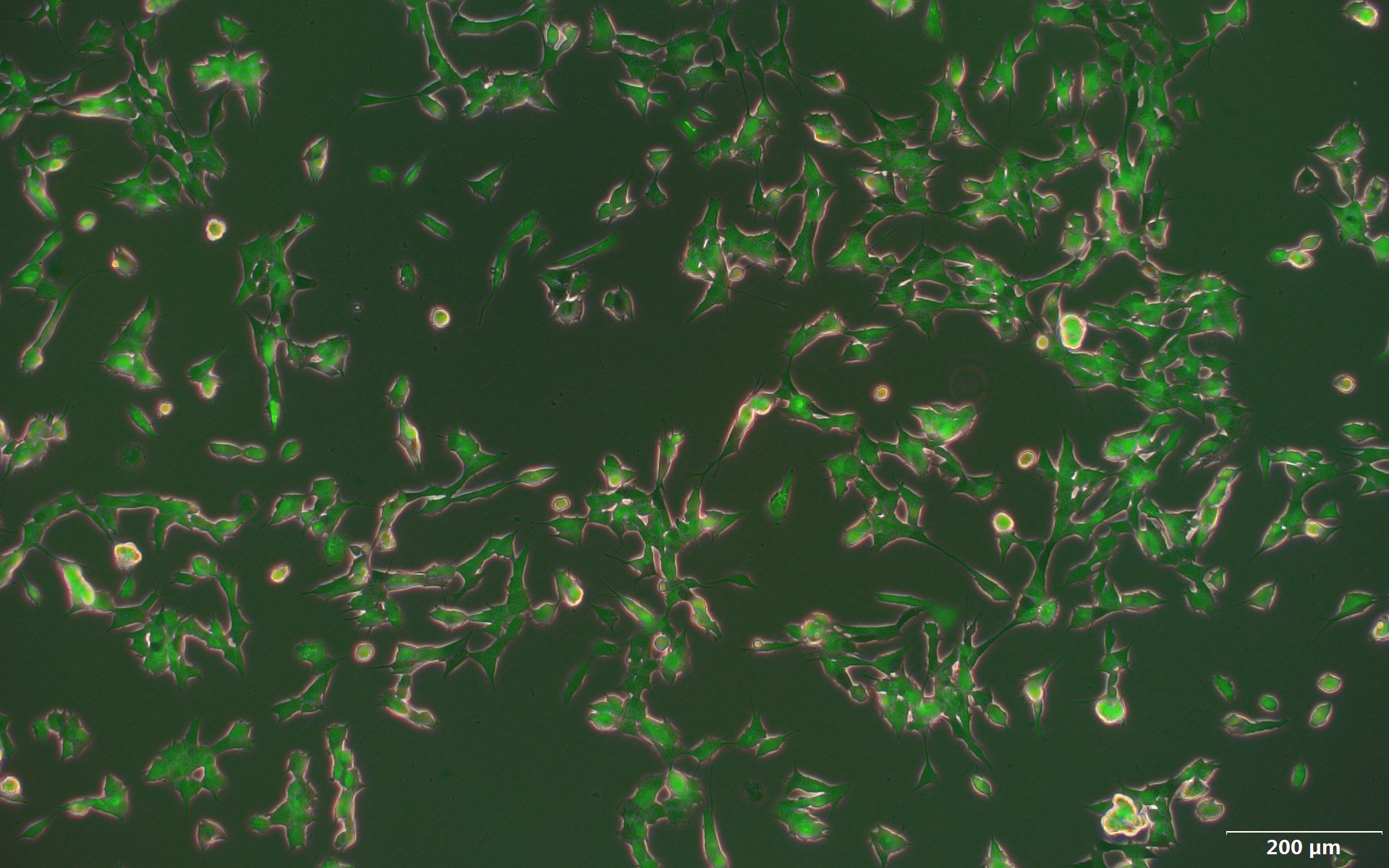

Supplement: Supplementary file 1 [file Data_Sheet_1.ZIP › raw data/fig4/fig4e/SH-SY5Y-APP-LV-miR-3940-5p-pre-merge.jpg]

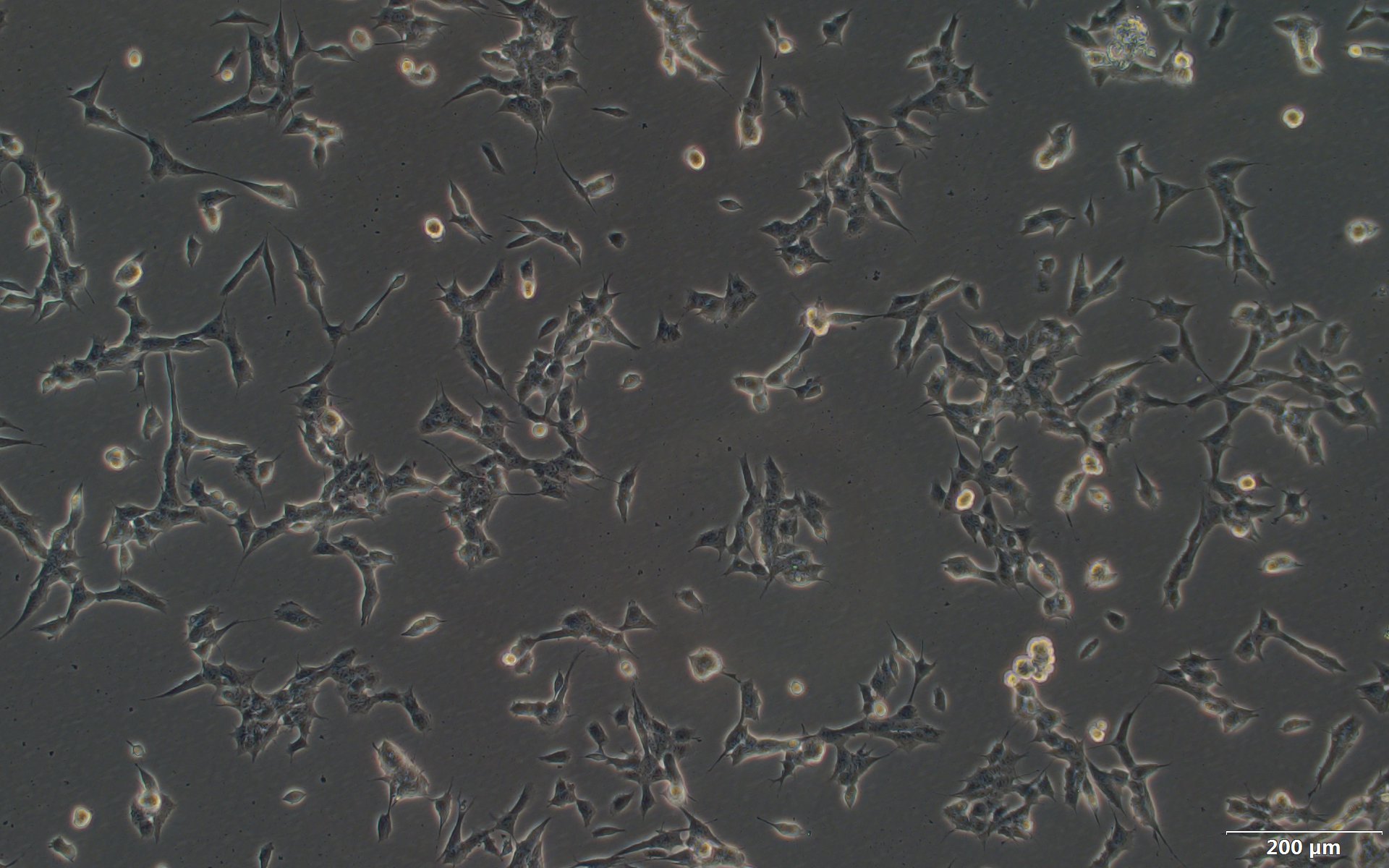

Supplement: Supplementary file 1 [file Data_Sheet_1.ZIP › raw data/fig4/fig4e/SH-SY5Y-APP-LV-miR-NC-BF.jpg]

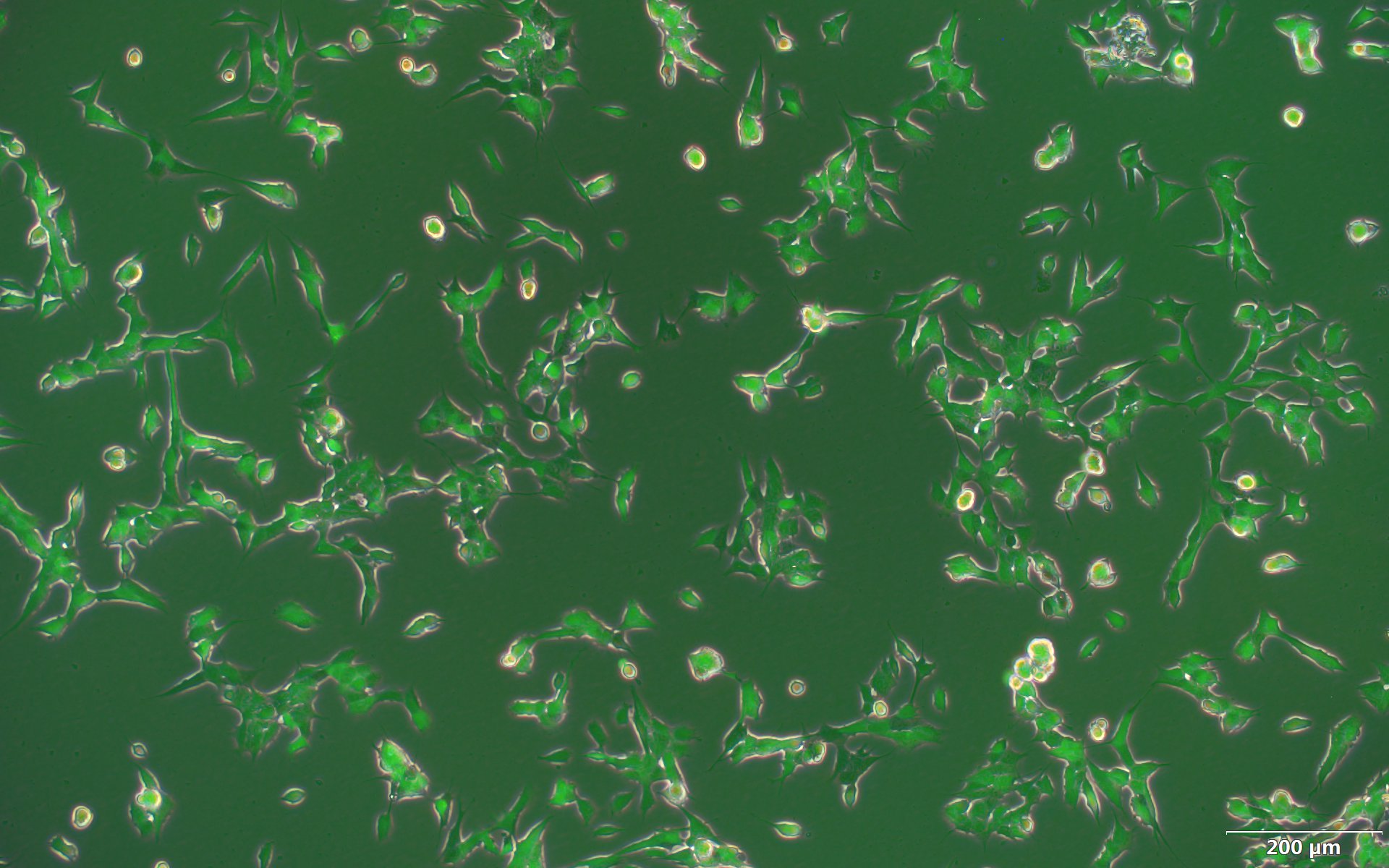

Supplement: Supplementary file 1 [file Data_Sheet_1.ZIP › raw data/fig4/fig4e/SH-SY5Y-APP-LV-miR-NC-Flu-merge.jpg]

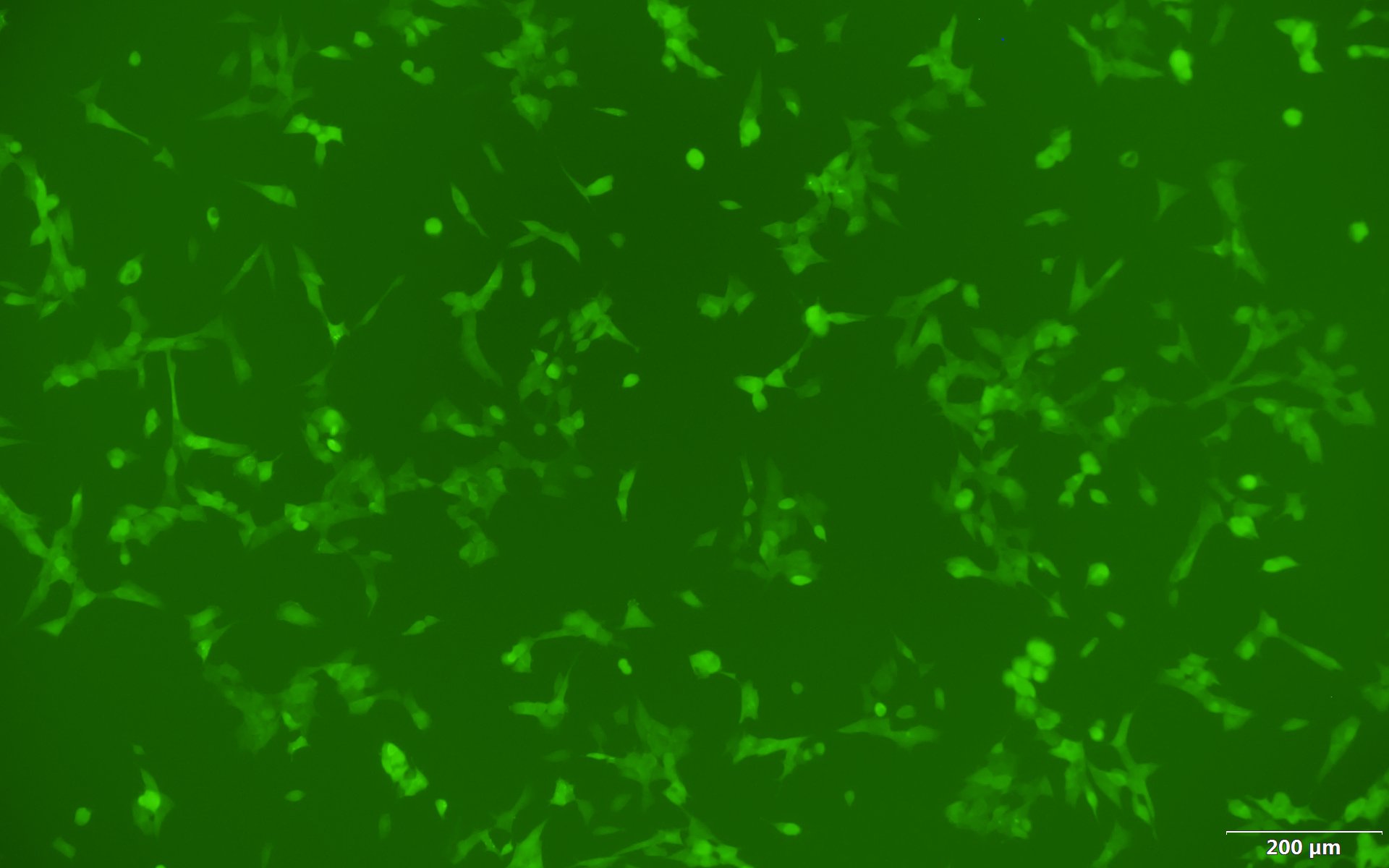

Supplement: Supplementary file 1 [file Data_Sheet_1.ZIP › raw data/fig4/fig4e/SH-SY5Y-APP-LV-miR-NC-Flu.jpg]

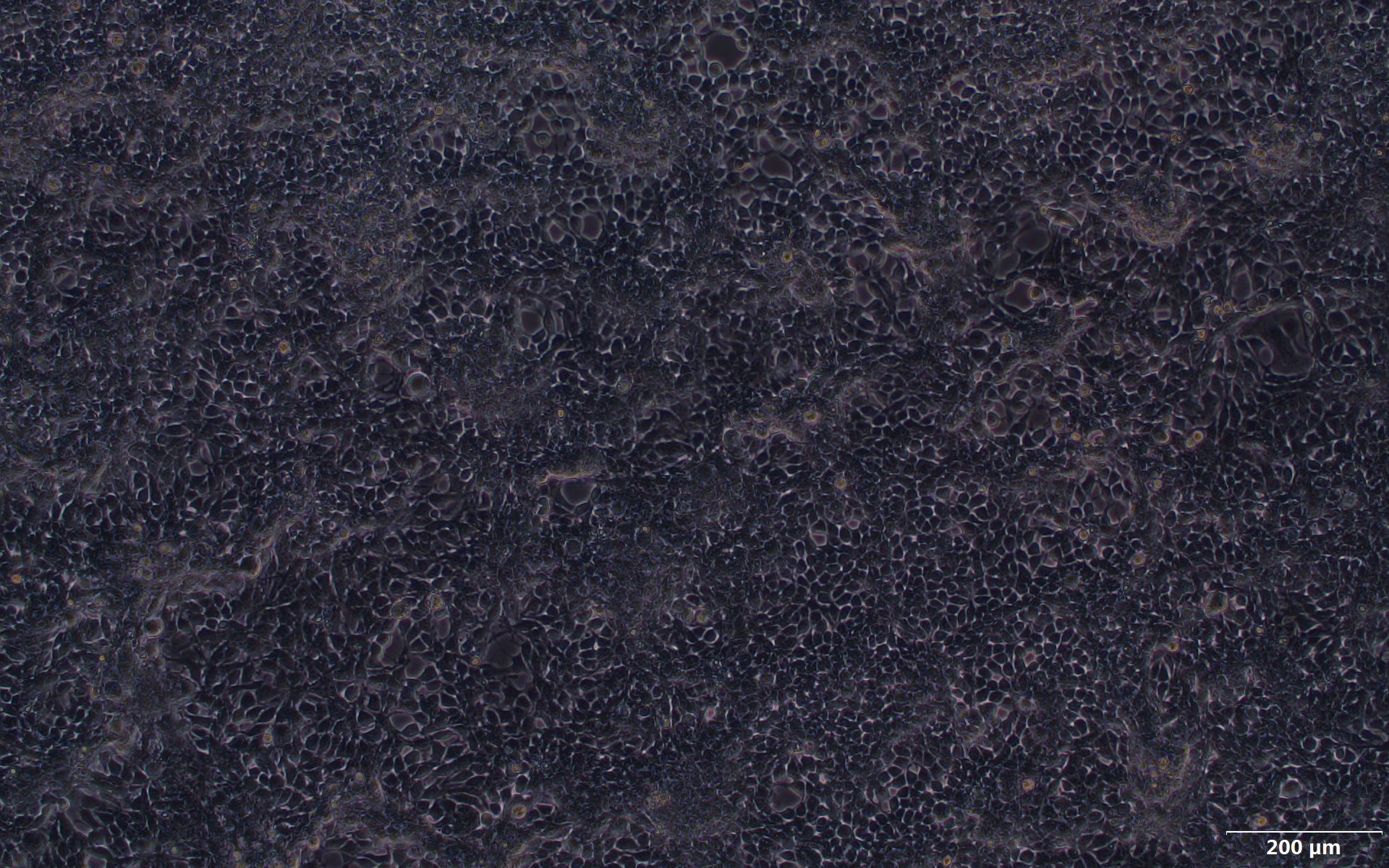

Supplement: Supplementary file 1 [file Data_Sheet_1.ZIP › raw data/fig4/fig4e/SH-SY5Y-APP-merge.jpg]

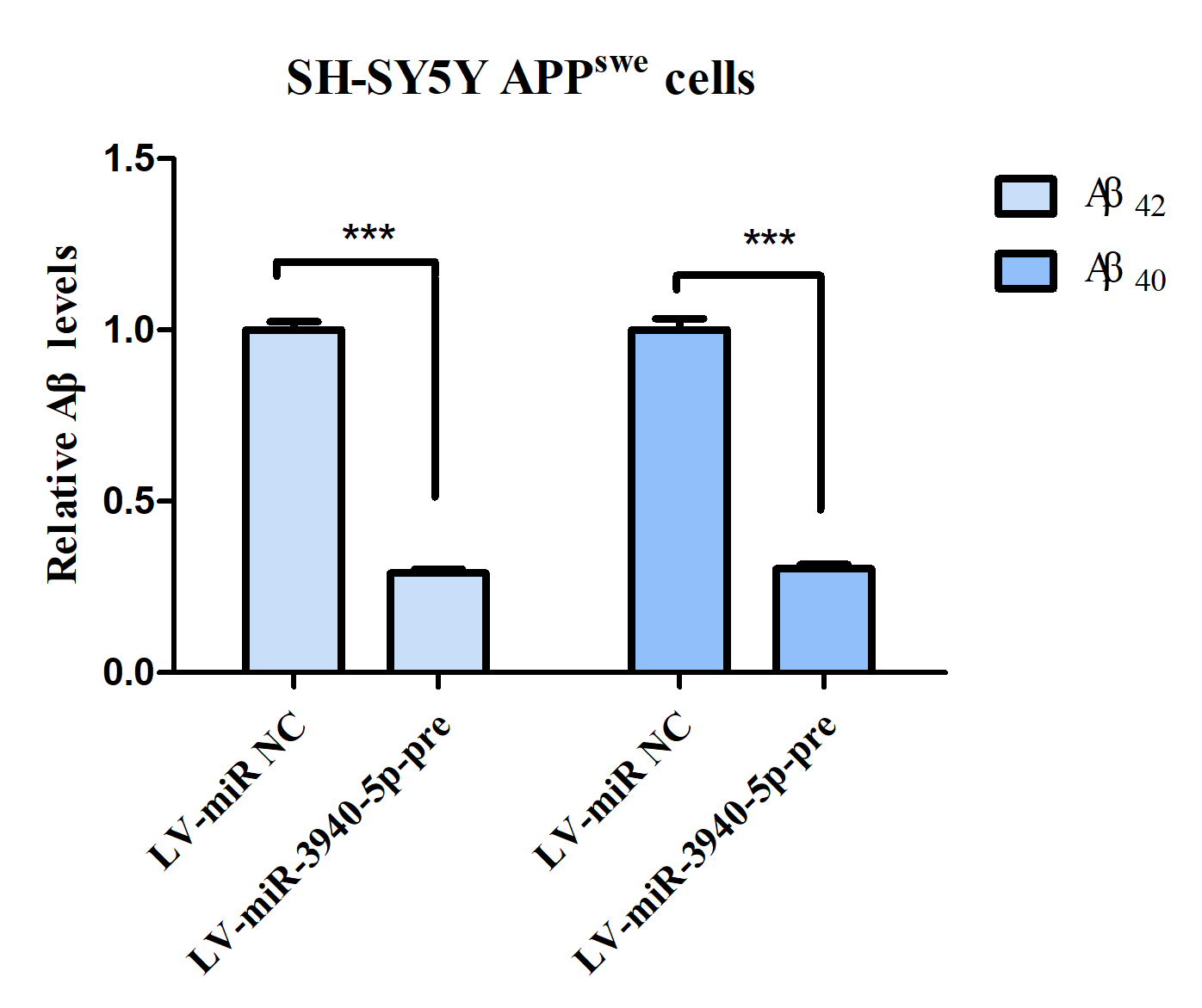

Supplement: Supplementary file 1 [file Data_Sheet_1.ZIP › raw data/fig4/fig4f-g/fig4f.tif]

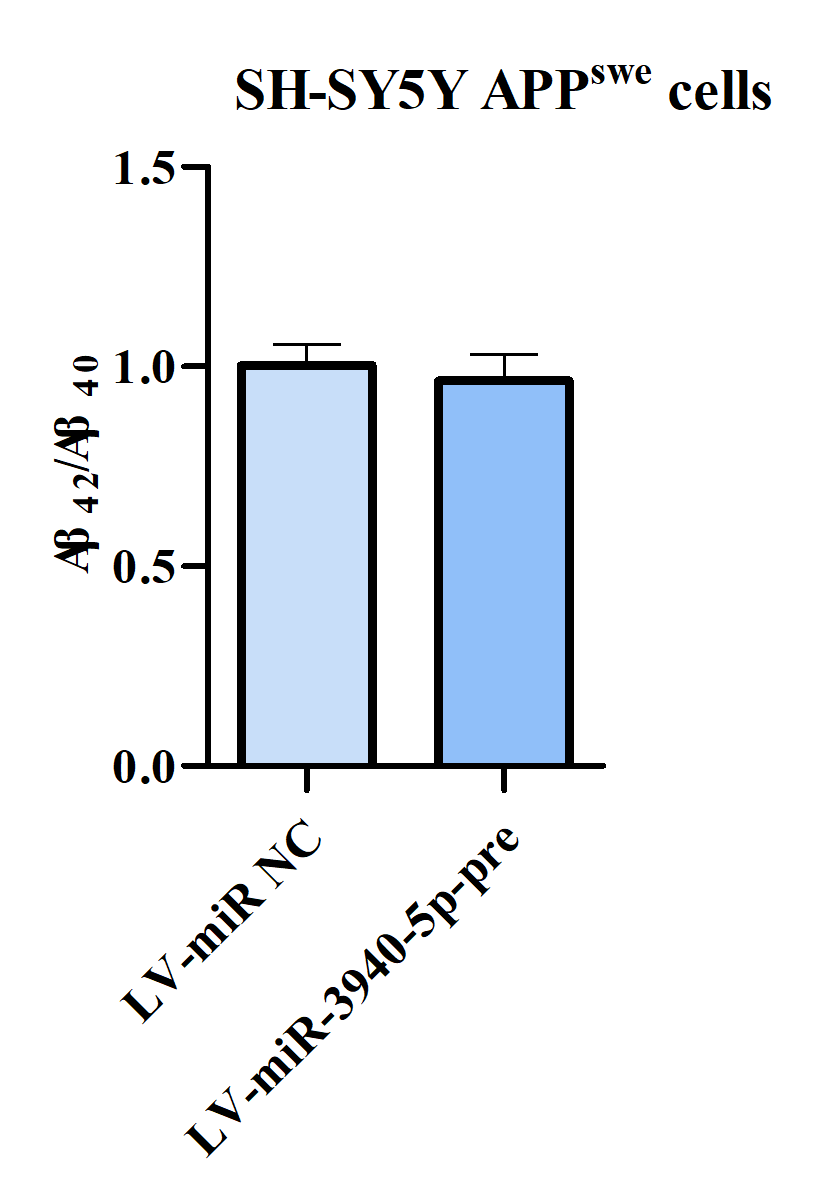

Supplement: Supplementary file 1 [file Data_Sheet_1.ZIP › raw data/fig4/fig4f-g/fig4g.tif]
